# Multiomics dissection of molecular regulatory mechanisms underlying autoimmune-associated noncoding SNPs

**DOI:** 10.1172/jci.insight.136477

**Published:** 2020-09-03

**Authors:** Xiao-Feng Chen, Ming-Rui Guo, Yuan-Yuan Duan, Feng Jiang, Hao Wu, Shan-Shan Dong, Xiao-Rong Zhou, Hlaing Nwe Thynn, Cong-Cong Liu, Lin Zhang, Yan Guo, Tie-Lin Yang

**Affiliations:** Key Laboratory of Biomedical Information Engineering of Ministry of Education and Biomedical Informatics & Genomics Center, School of Life Science and Technology, Xi’an Jiaotong University, Xi’an, China.

**Keywords:** Autoimmunity, Genetics, Autoimmune diseases, Bioinformatics, Genetic variation

## Abstract

More than 90% of autoimmune-associated variants are located in noncoding regions, leading to challenges in deciphering the underlying causal roles of functional variants and genes and biological mechanisms. Therefore, to reduce the gap between traditional genetic findings and mechanistic understanding of disease etiologies and clinical drug development, it is important to translate systematically the regulatory mechanisms underlying noncoding variants. Here, we prioritized functional noncoding SNPs with regulatory gene targets associated with 19 autoimmune diseases by incorporating hundreds of immune cell–specific multiomics data. The prioritized SNPs are associated with transcription factor (TF) binding, histone modification, or chromatin accessibility, indicating their allele-specific regulatory roles. Their target genes are significantly enriched in immunologically related pathways and other known immunologically related functions. We found that 90.1% of target genes are regulated by distal SNPs involving several TFs (e.g., the DNA-binding protein CCCTC-binding factor [CTCF]), suggesting the importance of long-range chromatin interaction in autoimmune diseases. Moreover, we predicted potential drug targets for autoimmune diseases, including 2 genes (*NFKB1* and *SH2B3*) with known drug indications on other diseases, highlighting their potential drug repurposing opportunities. Taken together, these findings may provide useful information for future experimental follow-up and drug applications on autoimmune diseases.

## Abstract 2

This systematic analysis provides insights into noncoding susceptibility SNPs associated with 19 autoimmune diseases across multiple regulatory levels, including SNP functionality, gene targets, regulatory mechanisms underlying SNPs, and gene function.

## Introduction

Autoimmune diseases are groups of complex immune system disorders with high prevalence rates worldwide (4.5%) (1). High heritabilities have been previously observed on various autoimmune diseases (~60%–90%) (2). In addition, GWAS have unraveled hundreds of susceptible loci associated with autoimmune diseases (3), suggesting many functional genes involved in some key immunological pathways (e.g., *MHC* gene clusters in antigen presentation, *TYK2* in cytokine signals) (4). However, the true functional variants and target genes for most GWAS variants remain largely unknown (4), and their discovery may be limited by 2 challenges. First, the detected variants may be in linkage disequilibrium (LD) with causal functional SNPs without genotyping. Second, more than 90% of GWAS variants are located in the uncultivated noncoding regions, complicating their functional interpretation.

Recent studies have integrated functional epigenetic data to predict noncoding SNP function. Many of these methods, such as CADD (5), DeepSEA (6), GWAVA (7), FATHMM-MKL (8), ReMM (9), and FIRE (10), adopted machine-learning algorithms to develop classifiers through integrating various annotations and labeled training data to distinguish potential functional and nonfunctional SNPs. However, the prior labeled training data may be both inaccurate and impractical owing to the current knowledge limitation in functional roles underlying noncoding SNPs. Other methods, such as RegulomeDB (11), 3DSNP (12), GWAS4D (13), IW-Scoring (14), Eigen (15), and FunSeq2 (16), either directly combined various epigenetic regulatory features to rank SNP functionality or adopted a weighted scoring scheme by considering the relative importance of each feature to assign SNP functionality scores. However, these approaches incorporated epigenetic or transcriptional annotation across all cells or tissues, omitting the cell- or context-specific regulation, aiming to prioritize potential functional variants rather than to dissect the downstream regulatory circuits linking functional variants to disease etiology. Autoimmune disease–associated variants are significantly enriched in blood cell-specific enhancers (17), implying that the integration of cell-specific functional data are required for dissecting molecular regulatory mechanisms underlying noncoding variants associated with autoimmune diseases.

The incorporation of cell-specific multiomics data has accelerated the decryption of functional mechanisms underlying noncoding GWAS variants (18, 19). Recently, we identified a functional SNP, associated with 2 autoimmune diseases, that exerted allele-specific enhancer regulation on *IRF5* expression through long-rang loop formation (20). Nevertheless, these studies primarily focused on one GWAS susceptibility loci on one disease, and only a limited number of functional causal variants predisposing to autoimmune diseases have been validated (20). The autoimmune diseases share substantial common susceptibility variants and immunopathology (21). Therefore, deciphering the functions of GWAS noncoding variants systematically is essential for accelerating GWAS findings into useful biological and clinical insight into the causes of autoimmune diseases.

To address these issues, we devised an integrative analysis frame to prioritize potential functional noncoding SNPs on 19 autoimmune diseases and further predicted their local and distal regulatory target genes using epigenetic, transcriptional, and 3D chromatin interaction data across hundreds of blood immune cell types (Supplemental Tables 2, 3, and 6; supplemental material available online with this article; https://doi.org/10.1172/jci.insight.136477DS1). Our analysis contains an integrative functional SNP prioritization method combing cell-specific epigenetic scoring and allele-specific regulatory analysis. We next evaluated the performance of our method by comparing it with other representative methods. We then explored the immunologically related function as well as potential clinical drug applications for predicted target genes. In addition, we analyzed the roles of long-range chromatin interactions on autoimmune SNPs as well as potential key regulatory transcription factors (TFs). Finally, we developed an open web resource (http://fnGWAS.online/) and a local analytical pipeline (https://github.com/xjtugenetics/fnGWAS).

## Results

### Integrative analysis prioritized potential noncoding functional autoimmune SNPs with causal target genes.

We collected 18,857 autoimmune noncoding tag SNPs predisposed to 19 distinct autoimmune diseases (reported *P* < 5×10^–8^) from multiple resources (3, 22, 23) (Supplemental Table 1). LD analysis retained 51,594 noncoding tags and LD-expanded (*r*^2^ > 0.8 in European ancestry) SNPs in 333 genome-wide significant loci (autoimmune-positive SNPs). We next collected 26,922,878 background SNPs in all 333 loci, and collected 47,131,427 negative SNPs beyond these loci (Supplemental Figure 1, further details in Methods). To explore potential key epigenetic regulatory features for autoimmune diseases, we collected 606 epigenetic annotation data across 47 blood immune cell types from Roadmap (24) and ENCODE Project (25) (Supplemental Table 2). Previous studies suggested that the autoimmune causal SNPs are significantly enriched in blood cell–specific enhancer marks (17). However, we found that compared with background SNPs, the autoimmune positive SNPs are significantly enriched higher for 347 active epigenetic features (Bonferroni’s adjustment after the χ^2^ test, FC > 1, *P* < 0.05/606) across 40 blood immune cell types within 4 epigenetic categories, including 9 DNase I hypersensitive sites (DHSs), 75 active histone modifications (H3K4me1, H3K4me2, H3K4me3, H3k27ac, and H3K9ac), 167 active genomic segmentations (HMM-15, marked as active transcription or enhancer), and 96 TF-binding sites (TFBSs) (Supplemental Figure 2 and Supplemental Table 1).

**Figure 1:**
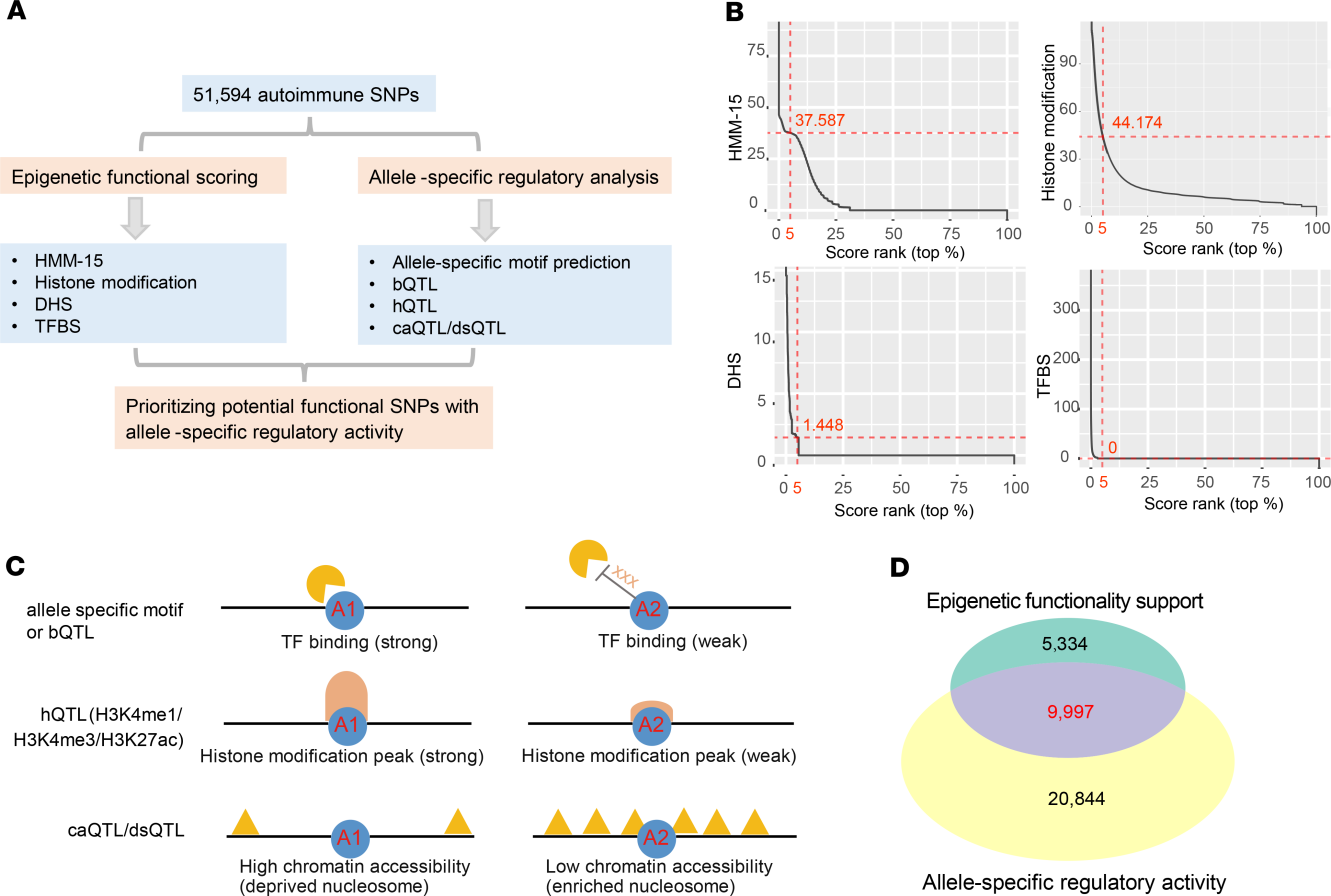
Prioritizing potential functional autoimmune noncoding SNPs. (**A**) Flowchart showing integrative analysis method for prioritizing potential functional autoimmune SNPs with allele-specific regulatory activities. See brief description for epigenetic scoring process in Supplemental Figure 1. See Methods for more detailed information. (**B**) Ranking plot for scores of all autoimmune negative SNPs within 4 epigenetic categories. Red dashed line represents top 5% ranked value. (**C**) Schematic showing several potential allelic molecular-level regulatory mechanisms underlying functional autoimmune SNPs. Multiple intermediate molecular quantitative trait loci (QTL) data in blood immune cell types were collected, including bQTL (transcription factor binding quantitative trait loci) (28), hQTL (histone modification quantitative trait loci), caQTL (chromatin accessibility quantitative trait loci) (31, 89, 90), and dsQTL (DNase-I hypersensitivity quantitative trait loci) (30, 87). See more description for each QTL data in Supplemental Table 3. (**D**) Venn diagram showing overlapping of autoimmune SNPs with predicted allele-specific regulatory activity and autoimmune SNPs with at least one functionality support by the epigenetic functional scoring. The overlapped SNPs were prioritized to be potential functional.

To evaluate the functionality of all positive autoimmune SNPs, we applied a 2-step integrative analysis strategy (Figure 1A). First, we developed a new and improved epigenetic functional scoring system, based on our previous epigenetic enrichment method (26, 27), using fold enrichment of all 347 significant epigenetic features across 4 epigenetic categories as scoring weight (flowchart shown in Supplemental Figure 1, further details in Methods). We could therefore obtain 4 independent functional scores across 4 different epigenetic categories on each SNP. By comparing the scoring rank of each positive SNP among all negative SNPs, we prioritized 15,314 SNPs associated with 19 autoimmune diseases with functionality support on at least 1 epigenetic category (Figure 1B and Supplemental Table 4). Second, we incorporated allele-specific motif prediction and multiple molecular-level quantitative trait loci (QTL) resources from multiple blood immune cell types to prioritize SNPs with potential allele-specific regulatory activities (Figure 1C and Supplemental Table 3), including TF binding QTL (bQTL) (28), histone modification QTL (hQTL) (29), DNase-I hypersensitivity QTL (dsQTL) (30), and chromatin accessibility QTL (caQTL) (31). We found that most (63.5%) autoimmune SNPs with functionality support by the epigenetic functional scoring also had potential allele-specific regulatory activities, including 9080 SNPs with predicted allele-specific TF binding, 434 bQTL SNPs with allele-preferable binding on 5 TFs (JunD, NF-κB, PU.1, Pou2f1, and Stat1) in lymphoblastoid cell lines (LCLs), 542 hQTL SNPs associated with chromatin modification on either H3K4me1 (*n* = 163), H3K4me3 (*n* = 176), or H3K27ac (*n* = 322) in LCLs, as well as 1028 caQTL or dsQTL SNPs associated with chromatin accessibility in either naive or stimulus-specific macrophages (*n* = 541), CD4^+^ T cells (*n* = 79), or LCLs (*n* = 585) (Supplemental Table 5). Further analysis revealed significant enrichment for multiple allele-specific regulatory activities on those SNPs with functionality support by the epigenetic functional scoring (Supplemental Figure 3), including allele-specific binding motif (Fisher’s exact test, FC = 1.02, *P* = 0.005, Supplemental Figure 3A) and multiple molecular QTL association (bQTL, dsQTL, caQTL, and hQTL) (Fisher’s exact test, FC = 1.56 to ~3.37, *P* < 0.05; Supplemental Figure 3, B–E). Taken together, through integrating epigenetic functional scoring and allele-specific regulatory analysis, we prioritized 9719 potential noncoding functional autoimmune SNPs with allele-specific regulatory activities (Figure 1D and Supplemental Table 4).

To explore potential regulatory target genes for these prioritized functional SNPs, we first integrated cis-QTL association and 3D chromatin interaction analysis using multiple regulatory data for more than 30 blood cell types (Supplemental Table 6). We predicted potential target genes either directly regulated by prioritized SNPs within target gene promoter (1 kb surrounding transcription starting sites [TSSs]) or regulated by distal functional SNPs through 3D chromatin interactions. To further validate the potential causal genetic regulatory effect for predicted genes, we employed 2 colocalization methods (32, 33) to assess whether the detected GWAS signal and cis-QTL association shared the same causal variant (posterior probability PP4 > 0.8) using all collected cell-specific cis-QTL and autoimmune GWAS data sets (Supplemental Tables 1 and 6). This multiomics analysis strategy has been shown to be useful for identifying functional causal genes at GWAS risk loci by our previous studies (20, 34). We predicted 354 potential target genes regulated by 2794 prioritized functional SNPs, which are supported by both cis-QTL, chromatin interaction, and colocalization analysis (Supplemental Tables 7 and 8).

### Integrative method improves prioritizing functional autoimmune SNPs compared with other methods.

To further assess the performance of our integrative method combing epigenetic functional scoring and allele-specific analysis, we compared the functional support on multiple immune cell–associated regulatory evidence between SNPs prioritized by our method and 5 other functional scoring methods, including 3DSNP (12), FIRE (10), GWAS4D (13), IW-Scoring (14), and RegulomeDB (11). To ensure fair comparison, we extracted top-ranked SNPs under different functionality support by our method (Supplemental Table 4) with corresponding equal or approximately equal counts of top-ranked SNPs from other methods, which resulted in comparison with 2 methods under all functionality support and 3 methods under selected functionality support (Table 1, see Supplemental Methods for details).

**Figure 2:**
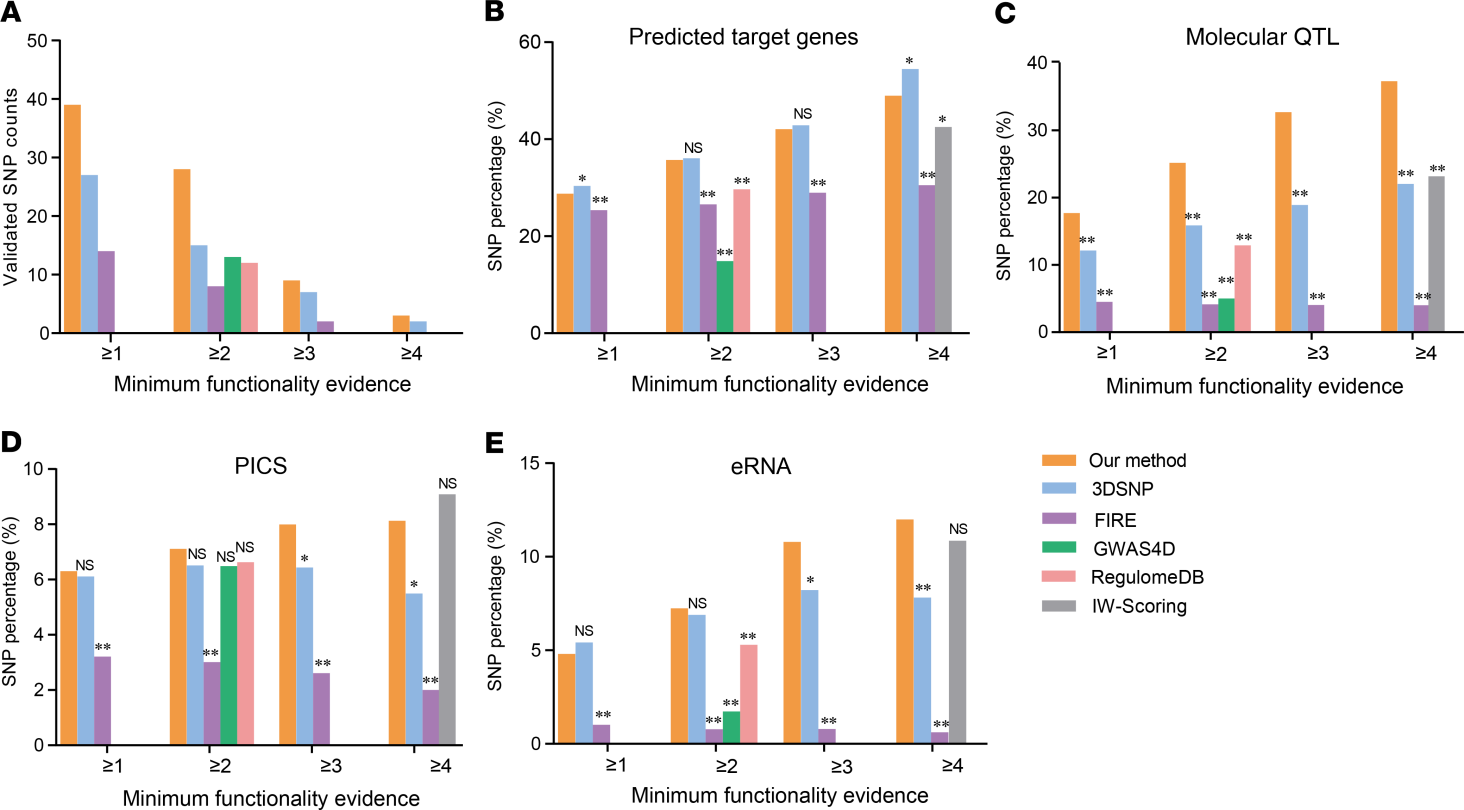
Comparing the integrative functional SNP prioritization method with other methods. (**A**) Comparison of experimentally validated functional SNPs between our method and 5 other methods from a high-throughput screen assay in mononuclear cells (35). (**B–E**) Comparison of percentage of annotated SNPs with different regulatory evidence between our method and 5 other methods, including (**B**) potential regulatory SNPs with predicted target genes by combining cis-QTL, chromatin interaction, and colocalization analysis (see Methods for detailed information), (**C**) potential functional SNPs with significant molecular QTL association on multiple blood immune cell types (Supplemental Table 3), (**D**) causal autoimmune- associated SNPs identified by PICS approach, and (**E**) SNPs annotated with enhancer RNA (eRNA). Fisher’s exact test was performed in **C–E** with the asterisk representing significant higher enrichment on our method (FC > 1). *P < 0.05, **P < 0.005. NS, not significant; PICS, Probabilistic Identification of Causal SNPs.

We first compared experimentally validated regulatory SNPs in mononuclear cells (35) and detected substantially more experimentally validated SNPs by our method compared with all 5 methods (Figure 2A). Consistent results were found in 2 nonimmune cell types (K562, HepG2) (36), in which our method had substantially more experimentally validated regulatory SNPs compared with 3 other methods (FIRE, GWAS4D, and IW-Scoring) (Supplemental Figure 4). In comparison with 3DSNP or RegulomeDB, we identified comparable experimentally validated SNPs in 2 nonimmune cell types with substantially more experimentally validated ones in the mononuclear cell, implying the potential outperformance of our method in prioritizing immune cell–specific regulatory SNPs. We next compared potential regulatory SNPs under multiple immune-related functional evidence (potential regulatory SNPs with predicted target genes, SNPs with significant molecular QTL association, causal SNPs identified by PICS approach [17], and enhancer RNA [eRNA] SNPs from FANTOM5 [37]). We found that our prioritized SNPs were enriched significantly higher for all functional evidence compared with FIRE (Fisher’s exact test, FC > 1, *P* < 0.05, Figure 2, B–E). We also detected much higher percentage of PICS causal SNPs (17) (Figure 2D) and significantly higher enrichment for all other functional evidence on our prioritized SNPs compared with either GWAS4D or RegulomeDB (Fisher’s exact test, FC > 1, *P* < 0.05, Figure 2, B, C, and E). In comparison with 3DSNP, we detected comparable percentage of regulatory SNPs with predicted target genes (Figure 2B) and significantly higher enrichment for all other functional evidence on our prioritized SNPs (Fisher’s exact test, FC > 1, *P* < 0.05, Figure 2, C–E). We also detected significantly higher enrichment for either regulatory SNPs with predicted target genes or molecular QTL SNPs (Fisher’s exact test, FC > 1, *P* < 0.05, Figure 2, B and C) and much higher percentage of eRNA SNPs (37) (Figure 2E) on our prioritized SNPs compared with IW-Scoring. Collectively, these analyses supported the outperformance in prioritizing functional autoimmune SNPs by our integrative analysis method over the other mentioned comparable methods.

### Allele-specific epigenetic regulatory effect mediated by risk alleles of functional autoimmune SNPs.

**Figure 3:**
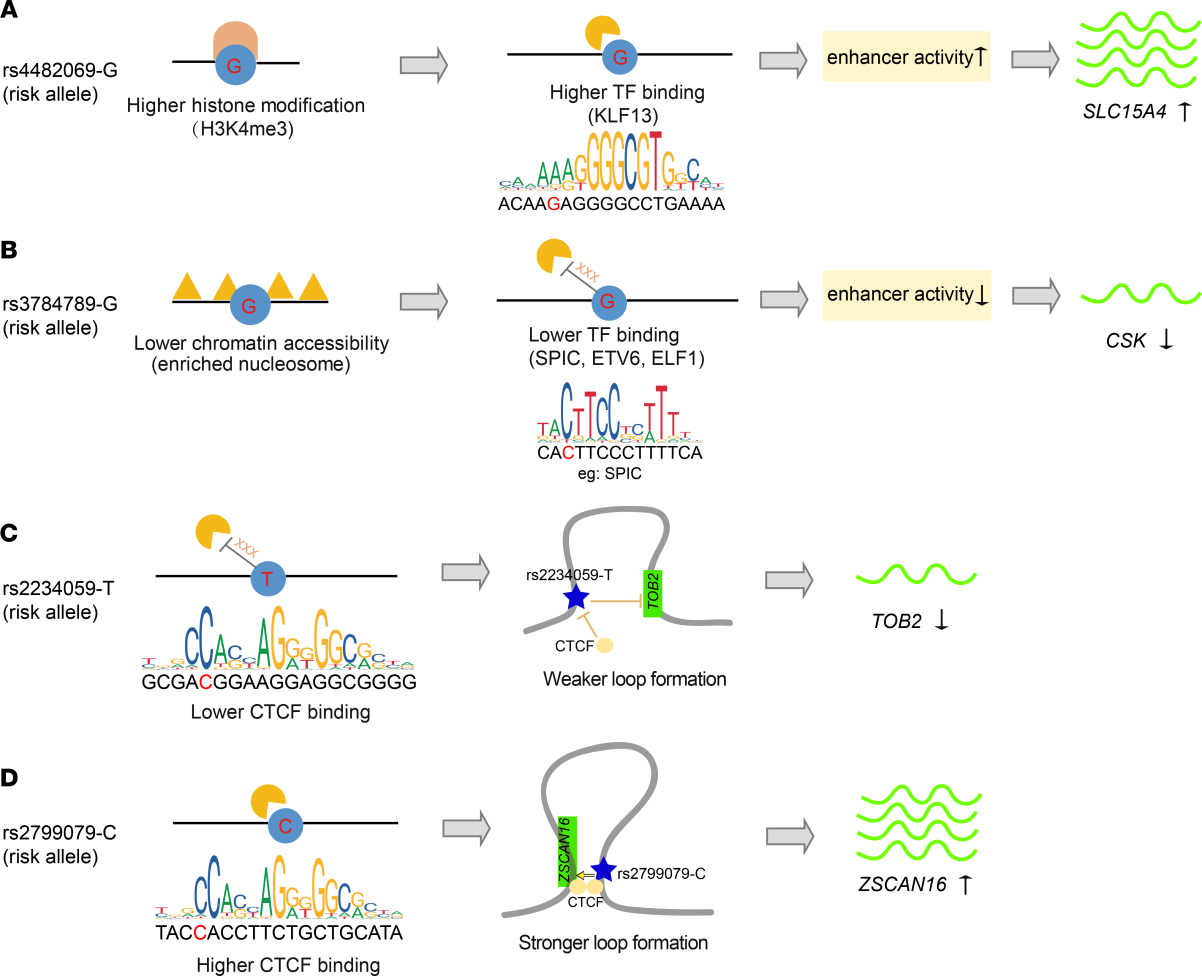
Allele-specific epigenetic effect mediated by risk alleles of prioritized SNPs. (**A**) Risk allele of rs4482069 is associated with higher histone modification (H3K4me3) in lymphoblastoid cell lines (LCLs), which may facilitate allele-specific binding of KLF1 and activate the enhancer activity to increase *SLC15A4* expression. (**B**) Risk allele of rs3784789 is associated with lower chromatin accessibility in LCLs, which may hamper binding of several TFs (SPIC, ETV6, and ELF1) and restrain the enhancer activity to decrease *CSK* expression. (**C** and **D**) Motif prediction revealed that CTCF had allele-specific binding to the nonrisk allele of rs2234059 (**C**) and risk allele of rs2799079 (**D**), respectively. The higher or lower CTCF binding may result in weaker (**C**) or stronger (**D**) long-range loop formation between SNP and target gene promoter, and further decrease (**C**) or increase (**D**) target gene expression, respectively. Detailed results for **A–D** appear in Supplemental Table 5 and Supplemental Table 7. More allele-specific regulatory examples are summarized in Supplemental Table 9.

The incorporation of multiple cell-specific multiomics data in our analysis may help decipher the allelic molecular mechanisms underlying prioritized noncoding functional SNPs. Figure 3, A–D, shows several SNPs in which the risk allele could exert allele-specific effect on target gene expression potentially through altering histone modification (H3K4me3, Figure 3A) or chromatin accessibility (Figure 3B) to affect the binding affinity of specific TFs, or directly modifying long-range chromatin looping potentially mediated by the DNA-binding protein CCCTC-binding factor (CTCF) (38) (Figure 3, C and D) to affect the proximity of distal regulatory enhancers. Supplemental Table 9 shows more regulatory circuits linking autoimmune SNP risk allele, allele-specific epigenetic regulatory effect (allele-specific TF binding or epigenetic modification or chromatin state), and regulatory effect on target gene expression.

### Target genes are significantly enriched in immunologically related functions.

**Figure 4:**
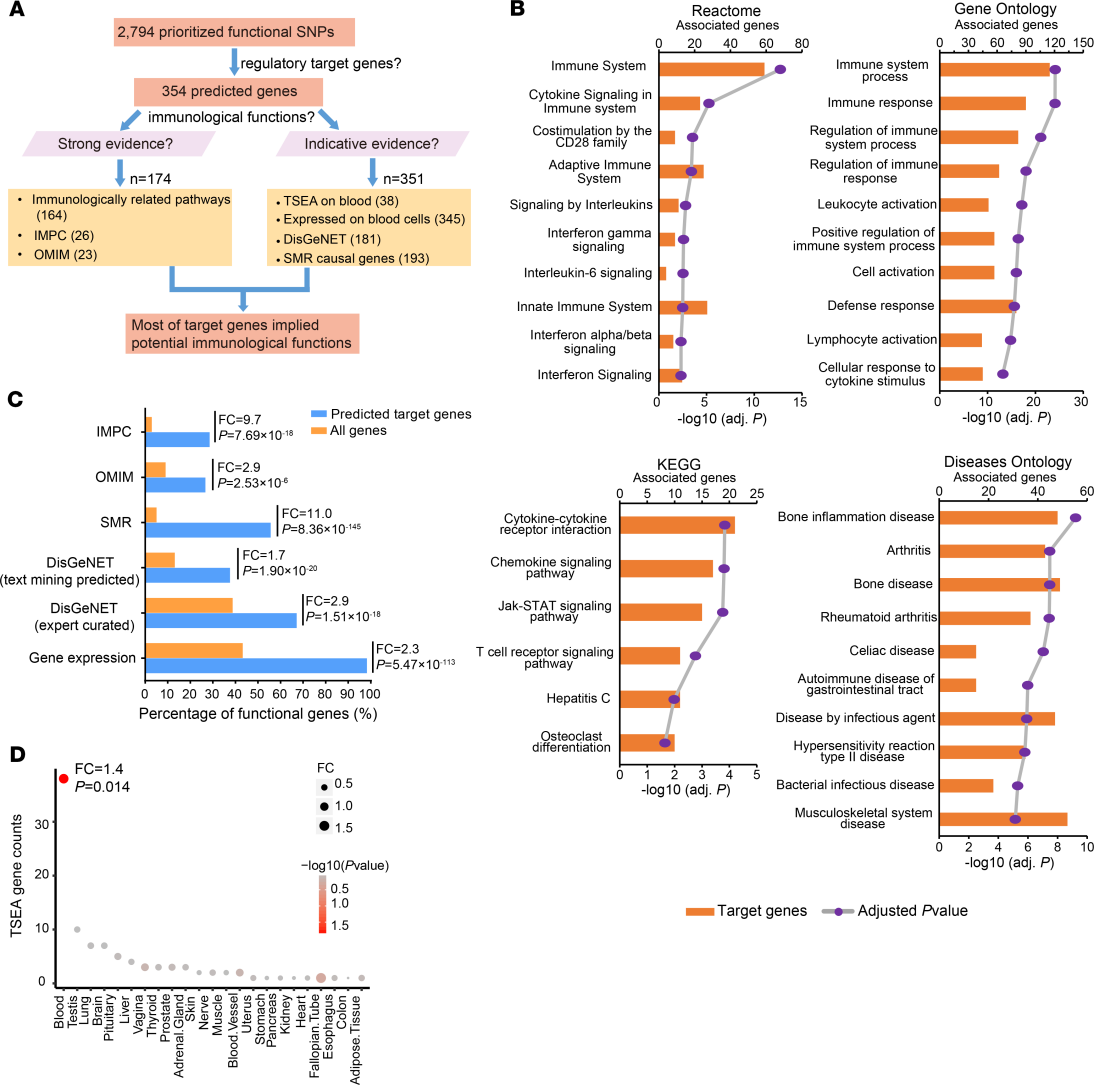
Immunological function analysis for predicted target genes. (**A**) Summary of multiple immunologically related functions for predicted target genes. (**B**) Top 10 most significant enriched biological pathways analyzed by clusterProfiler (43) on predicted target genes. Both *P* value (line chart) and gene counts (bar chart) are shown. (**C**) Functional enrichment for different immunologically related gene sets between predicted target genes and whole-genome genes. Multiple data resources were collected for gene functional analysis, including genes in which KO in mouse could cause abnormal immune system phenotypes collected from IMPC, genes associated with immunology-related Mendelian disorders collected from OMIM, potential causal GWAS effecter genes by SMR analysis (42), expert curated or text mining predicted immune system disease–associated genes from DisGeNET databases, and genes expressed (RPKM > 1) in blood immune cell types. Further description for each data resource is summarized in Supplemental Table 10. See Methods for detailed information. Enrichment analysis was performed using Fisher’s exact test. (**D**) Tissue Specific Expression Analysis (TSEA) for predicted target genes on 25 diverse tissues, with dot size representing gene counts and dot color indicating significance level (*P* value) using Fisher’s exact test. Only 1 significant (*P* < 0.05) tissue (blood) was detected.

To evaluate the immunologically related functions on 354 predicted target genes, we collected multiple immune-relevant functional data sets (Supplemental Table 10), including genes involved in immune-relevant pathways, genes in which KO in mouse could cause abnormal immune system phenotypes collected from the International Mouse Phenotyping Consortium (IMPC), as well as genes associated with immunology-related Mendelian disorders collected from the Online Mendelian Inheritance in Man (OMIM). Any genes annotated by the preceding 3 resources may indicate potential highly supported immune-relevant function. We identified 174 such genes (Figure 4A and Supplemental Table 11), including 164 genes participating in multiple immunologically related pathways, 26 genes in which KO in mouse could display abnormal immune system phenotypes from IMPC, as well as 23 genes associated with Mendelian disorders with immunology-related clinical symptoms from OMIM. We further analyzed other suggestive immune-relevant functions on predicted target genes (Supplemental Table 10), including genes with tissue-specific expression on blood, as determined by the Tissue Specific Expression Analysis (TSEA) approach (pSI < 0.01) (39), genes expressed in any blood immune cell types collected from either Roadmap (24) or DICE project (40) (RPKM > 1), expert curated or text mining predicted immune system disease–associated genes from DisGeNET databases (41), as well as potential causal GWAS effecter genes as determined by the summary data–based Mendelian randomization (SMR) analysis (42). We found that nearly all (351 of 354) target genes had suggestive immune-relevant function (Figure 4A and Supplemental Tables 11 and 12), including 345 genes expressed on blood immune cell types, 38 genes with tissue-specific expression on blood as determined by TSEA approach (pSI < 0.01) (39), 181 genes associated with immune system diseases collected from the DisGeNET database (41), as well as 193 genes with causal relationship with autoimmune diseases as implemented by SMR analysis (FDR < 0.05, *P*_HEIDI_ > 0.05) (42). Collectively, these data indicate potential immunological function for most gene targets, which may suggest new mechanistic insight into autoimmune disease etiologies.

To further verify the immunological roles for predicted target genes, we performed functional enrichment analysis using clusterProfiler (43). We found that the predicted target genes are significantly enriched in multiple immunologically related pathways. Figure 4B shows the top 10 most significantly enriched pathways (from Reactome, Gene Ontology [GO], Kyoto Encyclopedia of Genes and Genomes [KEGG], and Disease Ontology (DO); FDR < 0.05). These significantly enriched pathways include 8 overlapping autoimmune diseases that we analyzed, and show substantial genes in these disease pathways are regulated by prioritized functional SNPs associated with the same autoimmune disease (Supplemental Figure 5), further supporting the crucial immunological roles of our predicted target genes. We also detected significant enrichment for other immunologically related genes from different functional data sets (IMPC, OMIM, and DisGeNET) and SMR causal genes as well as expressed genes on blood cell types on predicted target genes (Fisher’s exact test, FC: 1.7 to ~11.0, *P*: 2.53 × 10^–6^ to ~8.36 × 10^–145^, Figure 4C). We further compared tissue-specific expression on 25 distinct cell types from TSEA (39), and only detected significantly higher enrichment for blood tissue (Fisher’s exact test, FC = 1.4, *P* = 0.014, Figure 4D) on predicted target genes, which also showed the largest number of tissue-specific expressed genes (Figure 4D). Altogether, these analyses revealed extensive enrichment of immunologically related functions for target genes, supporting the credibility of our target gene prediction.

### Prevailing long-range regulation linking functional autoimmune SNPs to distal target genes.

**Figure 5:**
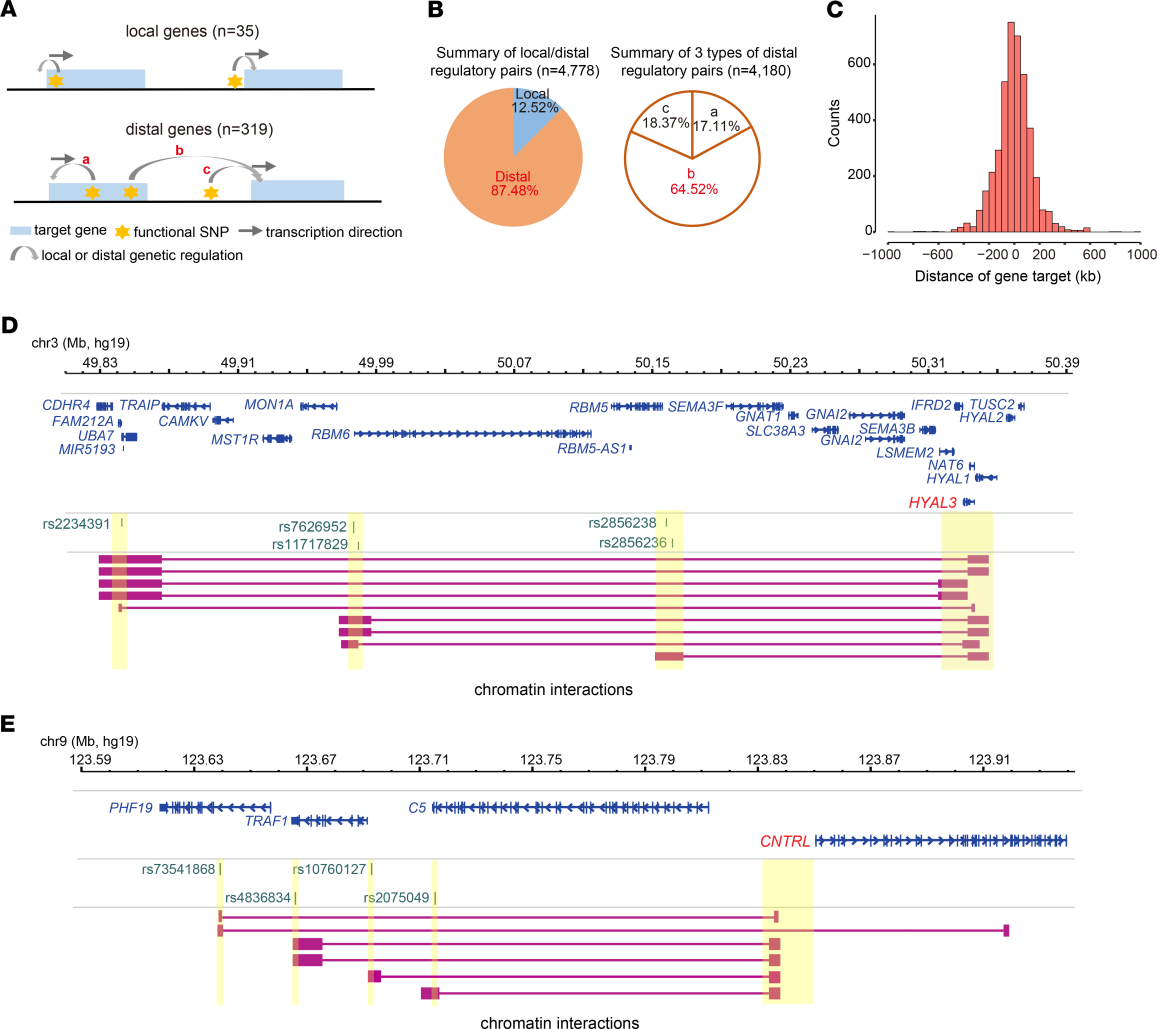
Prevailing long-range regulation linking functional SNPs to distal gene targets. (**A**) Schematic showing different regulatory models underlying prioritized functional autoimmune SNPs and gene targets. Local gene was defined as those exclusively regulated by functional SNPs within target gene promoter region (1 kb surrounding TSS). (**B**) Pie chart showing comparison between local and distal regulatory pairs (left), as well as between 3 types of distal regulatory pairs (right) in **A**. Local pair was defined as gene regulated by prioritized functional SNPs within target gene promoter region (1 kb surrounding TSS). (**C**) Counts of SNP-gene pairs at different distance (kb). (**D** and **E**) Two examples showing multiple functional autoimmune SNPs regulating distal target gene with (**D**) or without (**E**) known immunological function via long-range chromatin interactions. Functional evidence supporting SNP-gene regulatory relationship, including both chromatin interactions, cis-QTL association, and colocalization between GWAS association and cis-eQTL association (selected colocalization results are shown in Supplemental Figure 7). Genomic annotation and chromatin interaction were visualized using WashU Epigenome Browser. More example genes are shown Supplemental Figures 8–12.

The SNP-gene regulatory pairs could be divided either local genes (SNPs located within the target gene promoter) or distal genes (SNPs located outside the target gene promoter) (Figure 5A and Supplemental Table 7). Similarly, all regulatory target genes could be divided into local genes exclusively regulated by functional SNPs within the target gene promoter or distal genes that could be regulated by distal functional SNPs beyond the target gene promoter. We found that the definition of different-sized promoter region (from 1–10 kb surrounding TSS of target gene) had a negligible effect on both target gene prediction and proportion of distal and/or local target genes (Supplemental Figure 6). Therefore, we selected a stringent promoter definition (1 kb surrounding TSS) (44) for the following analysis. We detected a larger amount of distal genes (*n* = 319) compared with local genes (*n* = 35), including 148 distal genes exclusively regulated by distal functional SNPs (Figure 5A and Supplemental Table 7). These exclusive distal genes included many known immunologic genes, such as *CD37* (45), *CD28* (46), *IL7* (47), *IL12RB1* (48), or *IL2RA* (49). Previous functional assays have demonstrated that some distal immune-relevant genes could be regulated by distal enhancers in immune cell types, such as *IL2RA* (50), *CD58* (51), *IRF1* (52), or *IRF5* (20), indicating the important roles of long-range regulation on autoimmune diseases. We further analyzed all 4,778 SNP-gene regulatory pairs and detected predominantly distal pairs (87.48%) compared with local genes (Figure 5B and Supplemental Table 7). The prevailing long-range regulation may indicate that, for many functional noncoding autoimmune SNPs, their located or directly mapped genes might not be the direct regulatory target genes. Notably, the distal SNPs residing within local genes are more likely to regulate the distal target genes compared with their directly located genes (64.52% vs. 17.11%, Figure 5B). We also analyzed the distance between all distal regulatory pairs, and found that vast amount of distal SNP-gene regulatory pairs (65.23%) are located more than 50 kb away (mean distance: 104.07 kb, Figure 5C). Together, these analyses underscored the important roles of chromatin looping on autoimmune diseases.

### Many target genes are exclusively regulated by distal functional SNPs.

We further analyzed target genes exclusively regulated by distal functional SNPs, which may suggest some new important functional genes missed by traditional GWAS risk gene mapping strategy (located or nearest known disease relevant genes). We identified 128 such genes (Supplemental Table 7), including some known immunologically relevant genes and many other genes with unknown immunological roles. Figure 5D shows an immunologically relevant gene (*HYAL3*), in which multidimensional evidence (cis-QTLs, 3D chromatin interactions, and colocalization, Supplemental Tables 6 and 8 and Supplemental Figure 7) supported that multiple upstream functional SNPs could regulate distal *HYAL3* expression through long-range chromatin interactions. *HYAL3* encodes an enzyme for hyaluronan degradation, an immune regulator with roles in inflammatory response (53). Functional analysis revealed that *HYAL3* is involved in multiple immunologically relevant pathways (e.g., response to cytokine, response to IL-1, Supplemental Table 11), thus providing plausible mechanistic insight linking GWAS risk SNPs at the *HYAL3* locus to autoimmune pathogenies. Supplemental Figure 8 shows additional well-known examples of immunologically relevant genes (*IL6ST*, *CD5*) exclusively regulated by distal functional SNPs. *IL6ST* encodes a receptor of IL-6, and its loss of mutation causes immunodeficiency and abnormal inflammatory responses (54). *CD5* is a well-known negative regulator of TCR and BCR signaling, with critical roles in protecting against autoimmunity (55). Figure 5E shows another example gene (*CNTRL*) currently with unknown immunological roles, which is exclusively regulated by multiple upstream functional SNPs through long-range chromatin interactions. Functional analysis revealed that *CNTRL* was expressed on 20 blood cell types and associated with several immune system diseases according to DisGeNET database (Supplemental Table 11), indicating its potential roles in autoimmune etiology. Supplemental Figures 9–12 show additional example genes with indicative immunologically functional clues (*FAM213B*, *GBAP1*, *ICMT,* and *ICAM5*) and exclusively regulated by distal functional SNPs. Together, these analyses suggest many new potential immunologically relevant genes without directly located functional SNPs, which may provide new mechanistic insight linking distal functional SNPs at GWAS risk loci to autoimmune pathogenies via long-range chromatin interactions.

### Distal autoimmune genetic regulatory network may be mediated by several key TFs.

**Figure 6:**
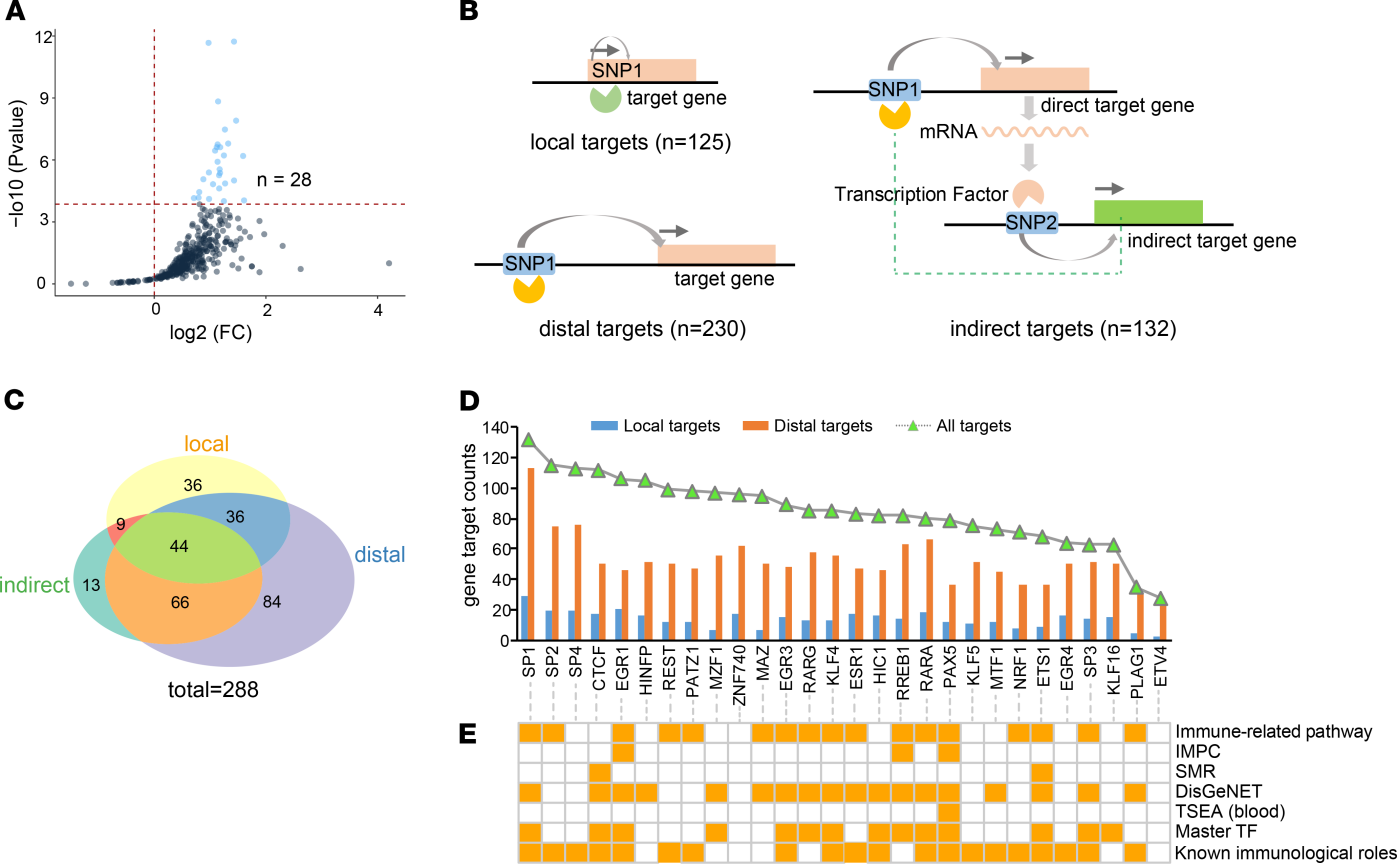
Identifying key TFs mediating autoimmune genetic regulatory network. (**A**) Scatter plot showing fold enrichment (FC) and significance enrichment level in 368 predicted motif TFs between prioritized functional SNPs and all autoimmune SNPs using Fisher’s exact test. Significantly higher enriched TFs (FC > 1, *P* < 0.05/368) are marked in blue. See detailed motif enrichment analysis results in Supplemental Table 13. (**B**) Schematic showing 3 TF-target gene regulatory models. The gray arrow indicates SNP-target gene interaction. (**C**) Venn diagram showing counts of 3 types of target genes on significant TFs in **B**. (**D**) Comparison between distal and local target genes on significant TFs. Count of all target genes are shown with TFs sorted by their count. (**E**) Annotated immunological functions on significant TFs (see detailed TF function information in Supplemental Table 13). The immunological functional resources are briefly described in Figure 4. Further detailed description is summarized in Supplemental Table 10. See Methods for detailed information.

To identify potential functional TFs mediating genetic regulation for autoimmune diseases, we compared allele-specific motifs occupying between 2794 functional SNPs with predicted target genes (Supplemental Table 7) and all autoimmune SNPs. We predicted 368 allele-specific motif TFs on functional SNPs, among which 28 TFs are significantly enriched higher for functional SNPs (Bonferroni’s adjustment after Fisher’s exact test, *P* < 0.05/368, FC > 1, Figure 6A and Supplemental Table 13). To explore potential regulatory targets on prioritized TFs, we considered 3 possible TF-gene regulatory models (Figure 6B), including (a) a local model, TFs directly bind to target gene promoter (1 kb surrounding TSS) to mediate gene expression; (b) a distal model, TFs bind to distal enhancers to regulate target gene expression via long-range chromatin interactions; and (c) an indirect model, the TFs regulate target gene expression through mediating other regulatory TFs in a trans manner. We found that most of predicted target genes (81.36%, 288 of 354) could be regulated by these 28 TFs (Figure 6, B and C; and Supplemental Table 13), with predominant distal model (*n* = 230) compared with either local model (*n* = 125) or indirect model (*n* = 132). Moreover, except for 3 Sp-family TFs (SP1, SP2, and SP4), CTCF had the most regulatory target genes (total = 112: local = 17; distal = 50; indirect = 70, Figure 6D and Supplemental Table 13), consistent with its known role in facilitating long-range chromatin looping (38). Further analysis revealed that all 28 TFs had more distal regulatory target genes compared with local genes (Figure 6D), and that 26 involved potential immunological roles according to multiple collected functional data sets (Supplemental Table 10) or previous literature reports (Figure 6E and Supplemental Table 13), implying their broad roles in distal genetic regulation on autoimmune diseases. We further analyzed the sharing of gene targets between different TFs, and detected 19 TFs sharing all target genes of 28 enriched TFs (Supplemental Figure 13). Most of these TFs (13 of 19) had known immunological roles according to previous studies (Figure 6E and Supplemental Table 13), including 10 master TFs (RREB1, RARA, HIC1, RARG, EGR3, ETS1, SP1, PAX5, MZF1, and CTCF) (56) in blood cell types, indicating their potential central regulatory roles for autoimmune diseases. Together, these analyses suggested several possible key regulatory TFs mediating distal genetic regulatory networks on autoimmune diseases.

### Analyzing potential clinical applications on target genes.

**Figure 7:**
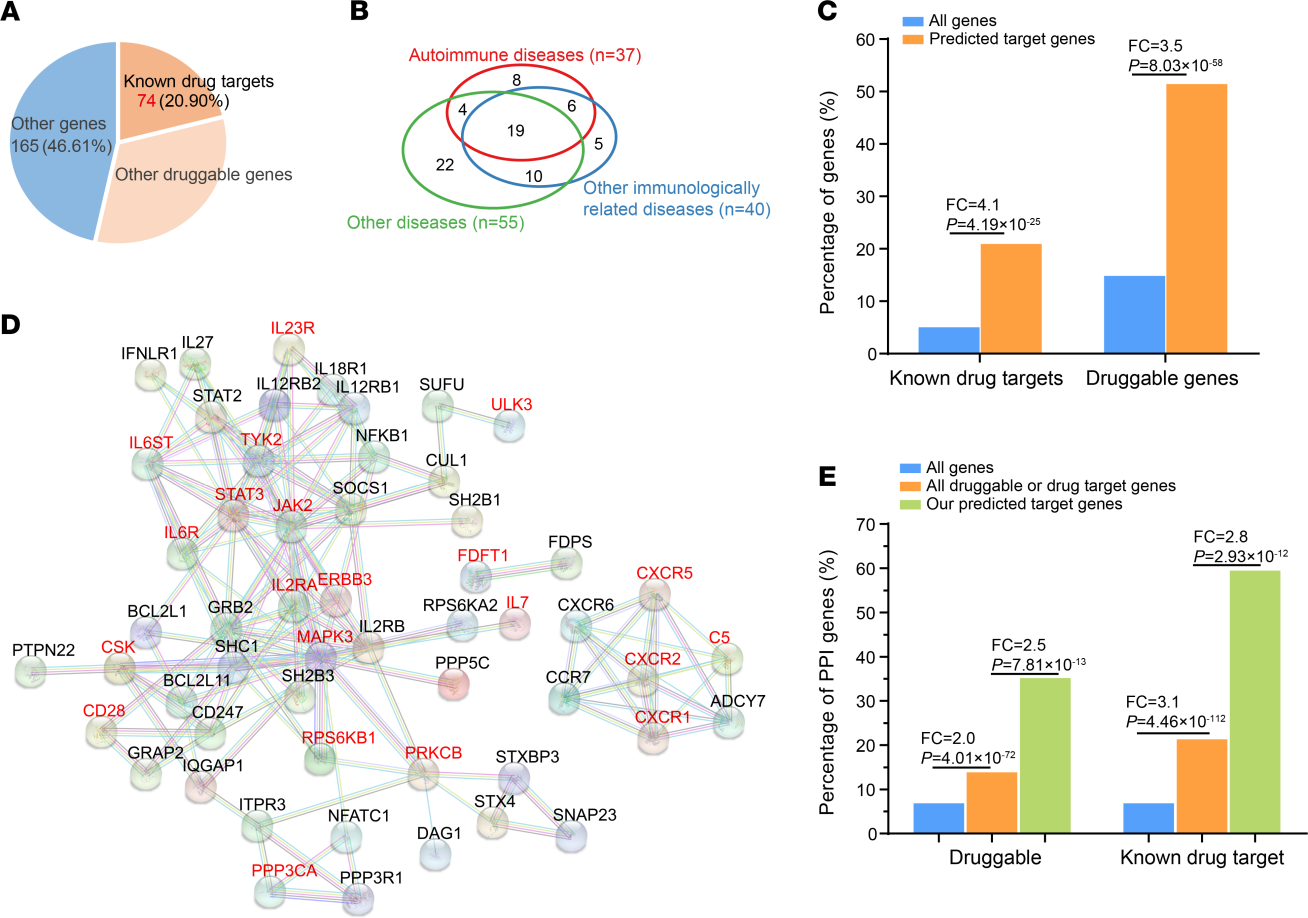
Drug implications analysis on predicted target genes. (**A**) Pie chart showing percentage of predicted target genes for either known drug target genes or predicted druggable genes or others. (**B**) Venn diagram showing sharing of drug target genes with indications on either autoimmune diseases, other immunologically related diseases or other diseases. See Supplemental Table 14 for detailed indication information and classification of these 3 disease types. (**C**) Functional enrichment analysis for either known drug target or predicted druggable genes on our predicted target genes compared with all genome genes using Fisher’s exact test. (**D**) PPI between autoimmune-drug target genes (marked in red) and other drug target or druggable genes. PPI plot was queried online from STRING database (score > 0.9). (**E**) Functional enrichment analysis showing percentage of genes with strong PPI (score > 0.9) with autoimmune-drug target genes on either predicted druggable genes or known drug target genes. The comparison was performed between predicted target genes (green) and all druggable or drug target genes (orange), as well as between all druggable genes or drug targets and all genome genes (blue) using Fisher’s exact test.

To explore potential clinical implications on predicted target genes, we first investigated all approved or experimental drug targets with known indications from multiple resources (57–62) (Supplemental Tables 14 and 15). We identified 74 genes targeted by drugs with known clinical indications on either autoimmune diseases (*n* = 37) or other immunologically related diseases (e.g., allergies, infections, or inflammations, *n* = 40) or other diseases (*n* = 55) (Figure 7, A and B, and Supplemental Table 14), implying the extensive therapeutic implications on predicted target genes. The identified drug target genes showed pervasive shared drug indications, with 62.5% of genes targeted for other immunologically related diseases and 41.8% of genes targeted for other diseases also shared targeted indications for autoimmune diseases (Figure 7B and Supplemental Table 14), indicating potential pleiotropic therapeutic effect among drug targets. Except for known drug target genes, we also identified 182 potential druggable genes, including 115 without known drug target indications (Supplemental Table 15 and Figure 7A). In comparison with all genome genes, our predicted target genes are significantly more enriched in both known drug target genes (Fisher’s exact test, FC = 4.1, *P* = 4.19 × 10^–25^) and predicted druggable genes (Fisher’s exact test, FC = 3.5, *P* = 8.03 × 10^–58^) (Figure 7C), further supporting the potential important clinical implications on them.

**Figure 8:**
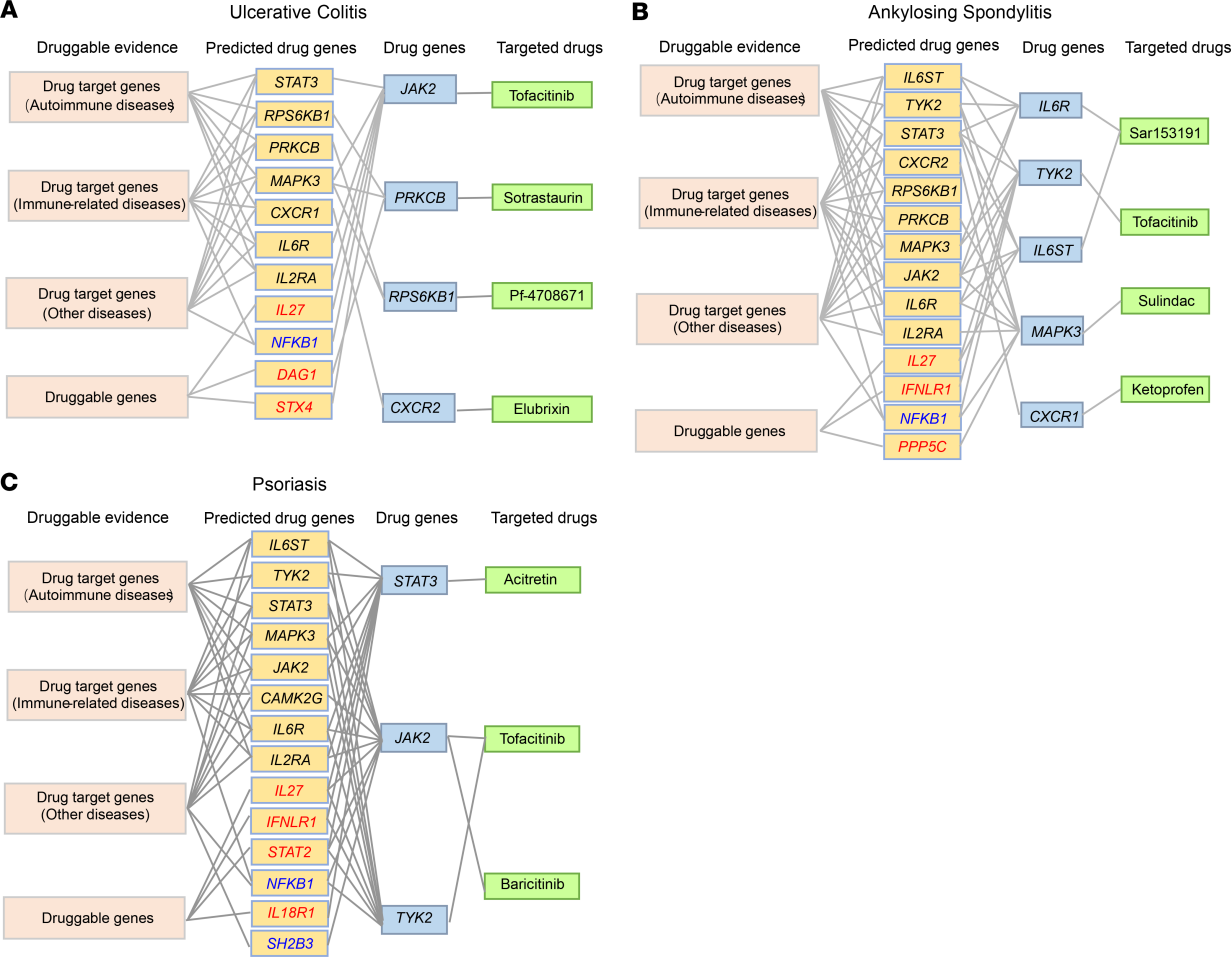
Predicted new targets with potential drug repurposing opportunities for autoimmune diseases. (**A–C**) Predicted new candidate drug targets on 3 autoimmune diseases. The orange rectangle shows predicted new drug genes. Genes with known indications on other autoimmune or nonautoimmune diseases are shown in black or blue. Genes without known drug target indications are shown in red. Supplemental Figure 15 shows predicted new candidate drug targets on 4 other autoimmune diseases. See Supplemental Table 16 for detailed drug indication information.

Consistent with the observed pleiotropic indications among drug target genes (Figure 7B), we found extensive disease association sharing for both autoimmune drug target genes and other drug target or druggable genes (Supplemental Figure 14, A and B), which may suggest new potential opportunities for drug repurposing on autoimmune diseases from other nonautoimmune drug target or druggable genes. To explore the functional relevance between known autoimmune-drug genes and other genes, we first analyzed their shared biological pathways. We found that most (32 of 37) autoimmune-drug genes shared the same immunologically related pathways with 68.7% (125 of 182) of drug target or druggable genes (Supplemental Figure 14C), implying their intimately functional connectivity. We further performed protein-protein interaction (PPI) analysis, and detected strong PPI (interaction score > 0.9) between 56.8% (21 of 37) of autoimmune-drug genes and 29 other known drug target or druggable genes (Figure 7D), indicating the pervasive regulatory relevance between known autoimmune-drug target and other genes. This was further supported by enrichment analysis, in which 37 autoimmune-drug genes showed significantly higher PPI with either predicted druggable genes (FC = 2.0, *P* = 4.01 × 10^–72^) or known drug target genes (FC = 3.1, *P* = 4.46 × 10^–112^) compared with whole-genome genes (Fisher’s exact test, Figure 7E). Moreover, when restricted PPI targets of autoimmune-drug genes to our predicted gene targets, we found significantly higher PPI on our predicted target genes compared with either all predicted druggable genes (FC = 2.5, *P* = 7.81×10^–13^) or all known drug target genes (FC = 2.8, *P* = 2.93 × 10^–12^) (Fisher’s exact test, Figure 7E). Based on these preceding analyses, it is reasonable to assume that incorporating both GWAS genetic regulation and protein-protein interaction network could help prioritize new potential drug target genes for autoimmune diseases. We prioritized 23 new candidate drug target genes for 7 autoimmune diseases (Figure 8, Supplemental Figure 15, and Supplemental Table 16), which showed both strong PPI with known drug target genes and genetic regulation associated with the same autoimmune disease. Among 23 prioritized genes, we found 14 genes with known drug indications on other autoimmune diseases as well as 2 genes (*NFKB1*, *SH2B3*) with indications on other diseases (Figure 8, Supplemental Figure 15, and Supplemental Table 16). The remaining 7 genes had no indications while with druggable evidence, including 3 genes (*DAG1*, *IL27*, *STX4*) predicted targeted for ulcerative colitis, 3 genes (*IL27*, *IFNLR1*, *PPP5C*) predicted targeted for ankylosing spondylitis, as well as 4 genes (*IFNLR1*, *IL27*, *STAT2*, *IL18R1*) predicted targeted for psoriasis (Figure 8 and Supplemental Table 16). Together, our analysis not only prioritized some new promising drug targets for future drug exploration, but also suggested some known drug targets (*NFKB1*, *SH2B3*) that could be exploited for future drug repurposing on autoimmune diseases.

### Open web application and local pipeline.

To facilitate quick searches for interested SNP(s) or gene(s) prioritized by our integrative analysis, we developed an open website (http://fngwas.online/) and collected comprehensive resources, including functional scores with predicted allele-specific regulatory activities on all noncoding autoimmune SNPs, regulatory target genes on prioritized functional SNPs, immunologically related functions for predicted target genes, and clinical drug applications for target genes. We also provided precomputed functional analysis results across whole-genome SNPs/genes for bulk downloading (http://fngwas.online/download.php), which included functional scores and predicted allelic regulatory mechanisms underlying all autosomal noncoding SNPs as well as multiple disease-relevant function and drug target analysis for all genome genes. To further expand the potential application of our analytical frame on other complex diseases and traits, we also developed packaged local pipeline named fnGWAS (dissecting the functionality of noncoding GWAS SNPs, workflow shown in Supplemental Figure 16), which could be run on any local Linux server with user-definable annotation data and parameters (https://github.com/xjtugenetics/fnGWAS). In our study, GWAS SNPs in European ancestry were selected and fnGWAS have provided built-in 1000 genome v3 genotype data in European samples (63). However, GWAS SNPs from the population of other ancestries (such as African) are also supported if the corresponding reference genotype data are prepared.

## Discussion

Most autoimmune susceptibility SNPs are located in the noncoding region. However, it remains challenging to pinpoint the causal SNPs and functional genes to decipher the underlying biological mechanisms. In this study, we systematically explored the molecular mechanisms underlying noncoding susceptibility SNPs associated with 19 autoimmune diseases, through combining functional SNPs scoring, allelic regulatory activity analysis, target gene prediction, gene function annotation, as well as drug application exploration. We found predominant long-range chromatin interaction linking functional SNPs to distal target genes, which may be mediated by several key TFs, including CTCF. Notably, we detected broad immunological functions and clinical drug applications on predicted target genes. In addition, we developed an open website and an analytical pipeline. Taken together, our study highlighted the intensive regulatory roles of noncoding SNPs associated with autoimmune diseases.

We have previously integrated epigenetic features for known disease-associated SNPs to predict novel susceptibility SNPs for complex diseases (26, 27, 64, 65). In this study, we developed a new and improved epigenetic functional scoring method together with allele-specific regulatory activity analysis to prioritize functional autoimmune SNPs through incorporating immune cell–specific active epigenetic information. Other comparable scoring methods have also been developed, such as 3DSNP (12), FIRE (10), GWAS4D (13), IW-Scoring (14), and RegulomeDB (11). Compared with these approaches, one distinct feature of our method was the integration of immune cell–specific epigenetic information (Supplemental Table 17), which may provide better evaluation for disease-specific functional autoimmune SNPs. Another feature of our analysis frame is the comprehensive functional evaluation on multiple regulatory levels spanning SNP functional scoring, analysis of allele-specific regulatory mechanisms underlying SNPs, gene target prediction, gene function analysis, as well as gene clinical application analysis (Supplemental Table 17). The integration of cell-specific epigenetic annotation has proven to be highly successful for prioritizing functional GWAS SNPs and validated by many recent experimental assays (20, 66). Our analysis revealed that the top-ranked autoimmune SNPs prioritized by our method are enriched significantly higher in multiple blood immune cell–associated regulatory elements compared with other methods, implying the outperformance of our method in prioritizing noncoding functional autoimmune SNPs.

Recent studies have shown that considerable noncoding GWAS SNPs could regulate target genes through long-range loop formation (67–69), providing unprecedented new mechanical insight underlying GWAS disease association. Consistently, our analysis revealed prevailing long-range regulation linking functional autoimmune SNPs to distal target genes, suggesting the important roles of chromatin interactions for autoimmune diseases. Our analysis also suggested that many functional SNPs within local genes could regulate distal target gene expression, including vast amounts of functional SNPs within local gene promoter. One underlying mechanism hypothesis was that the gene promoter could also act as an enhancer, to regulate distal gene expression (70), which was consistent with our recent findings that one functional autoimmune risk SNP within *TNPO3* promoter could independently regulate distal *IRF5* expression via long-range loop formation (20). We also identified several potential key regulatory TFs with significant enrichment in functional autoimmune SNPs, including CTCF. CTCF is well known for its regulatory roles for mediating enhancer-promoter interaction in chromatin loop formation (38), and played essential roles in late B cell differentiation (71). A previous study found that those downregulated genes after *Ctcf* knockdown in mouse T cell line are significantly enriched for immune-relevant pathways (72), further supporting the roles of CTCF in long-range regulation for autoimmune disease. In line with the prevailing long-range genetic regulation detected for autoimmune diseases, we also found predominant distal regulatory genes compared with local genes for all enriched TFs, indicating their potential roles in mediating distal genetic regulatory network for autoimmune diseases. Future functional assays are needed to decipher their precise regulatory mechanisms.

The past fruitful GWAS findings have remarkably accelerated the translation of new drug clinical utilities (73). The drug targets with human genetic evidence of disease association are twice as likely to lead to approved drugs (74). Consistently, we found that our predicted autoimmune target genes are significantly more enriched in both known drug target genes and druggable genes compared with whole-genome genes, supporting the potential important clinical implications on disease effecter genes. A previous GWAS study has incorporated PPI with 98 annotated RA risk genes to predict new drug targets, and highlighted *CDK6* and *CDK4* as promising candidates (75). The incorporation of functional genomic and immune-related annotations as well as PPI has been demonstrated successfully in prioritizing potential drug target on immune-related traits (76).Our study consistently integrated both genetic association and PPI, and prioritized 23 new candidate drug target genes on 7 autoimmune diseases, including many genes (16 of 23) with known indications on autoimmune diseases or other diseases. The drug repurposing strategies have shed light on many new promising therapeutic opportunities for autoimmune diseases, such as the dopaminergic drug for multiple sclerosis (77) or Fibrate for treating for primary biliary cirrhosis (78). Our results may provide important clues for future clinical drug repurposing on autoimmune diseases. For example, we predicted *IL2RA* to be a potential new drug target for ankylosing spondylitis. *IL2RA* is targeted by several known drugs (e.g., HuMax-TAC), with indications on autoimmune diabetes, and has known roles in the pathogenesis of autoimmunity (4). Moreover, we found that *IL2RA* was regulated by several functional SNPs associated with ankylosing spondylitis. Collectively, these data suggest the potential drug repurposing opportunity of *IL2RA* on ankylosing spondylitis.

## Methods

### Autoimmune SNPs collection

We collected SNPs associated with 19 autoimmune diseases (alopecia areata, ankylosing spondylitis, autoimmune thyroid disease, celiac disease, Crohn’s disease, IgE and allergic sensitization, inflammatory bowel disease, juvenile idiopathic arthritis, multiple sclerosis, narcolepsy, primary biliary cirrhosis, primary sclerosing cholangitis, psoriasis, rheumatoid arthritis, systemic lupus erythematosus, systemic scleroderma, type 1 diabetes, ulcerative colitis, and vitiligo) from multiple resources, including the GWAS Catalog (3), the ImmunoBase (https://www.immunobase.org/), and other public studies (22, 23). All databases were visited in March 2019 and summarized in Supplemental Table 1. For SNPs achieved genome-wide significance reported in European ancestry (*P* < 5×10^–8^), any coding or splicing SNPs annotated by ANNOVAR (79) using GENCODE v19 reference data were removed. We further excluded SNPs within the major histocompatibility complex locus (MHC) owing to the complex LD patterns. The filtered SNPs were selected as autoimmune tag SNPs.

### LD analysis, positive, background, and negative SNP definitions

LD analysis for autoimmune tag SNPs was conducted using PLINK v1.90 (80) in European samples from 1000 genome v3 genotype data (63), with maximum distance for *r*^2^ calculation set as 1 M. Genome-wide significant loci were defined as merged unique regions surrounding 1 M of any filtered noncoding tag SNPs with overlapping MHC region truncated. We extracted noncoding tags and LD expanded (*r*^2^ > 0.8) SNPs within genome-wide significant loci as positive SNPs and all noncoding SNPs in these loci as background SNPs. We collected 41,377 susceptible SNPs with ID record in the 1000 genome v3 genotype data (63) from the GWAS Catalog (visited in March 2019). All other noncoding SNPs beyond genome-wide significant loci and beyond the MHC region with low LD (*r*^2^ < 0.1) with the GWAS catalog-susceptible SNPs were selected as negative SNPs.

### Integrating epigenetic functional scoring and allele-specific regulatory activity analysis for prioritizing potential functional autoimmune SNPs

#### Epigenetic features selection.

We collected 606 epigenetic data (called peak region) on 47 blood cell types from Roadmap (24) and ENCODE Project (25) (Supplemental Table 2). Four different epigenetic categories of data were incorporated for SNP annotation, including 15 chromatin states (HMM-15), histone modification, DHSs and TFBSs. One epigenetic feature represents one epigenetic annotation in one cell type (e.g., H3K4me1 in GM12878). SNPs were labeled as annotated or unannotated on each epigenetic feature by analyzing their overlapping with selected feature using bedtools v2.25.0 (81). We performed enrichment analysis for each epigenetic feature by comparing counts of annotated positive SNPs and background SNPs using Χ^2^ test. All epigenetic features with significantly higher enrichment for positive SNPs compared with background SNPs (Fold enrichment > 1, Bonferroni’s adjusted *P* < 0.05) were selected for the following epigenetic scoring. The fold enrichment (FC) is defined as follows: FC = annotated positive SNPs × total background SNPs/annotated background SNPs × total positive SNPs.

#### Epigenetic functional scoring.

Based on our previous epigenetic enrichment approach (26, 27), we developed a new cell-specific epigenetic weighted scoring method to evaluate the functionality for all noncoding autoimmune positive SNPs (flowchart shown in Supplemental Figure 1). For each epigenetic category (HMM-15, histone modification, DHS, and TFBS), we adopted an accumulative quantitative score system using fold enrichment of selected significant features within each category as weight, separately, which is defined as follows:

 (Equation 1)
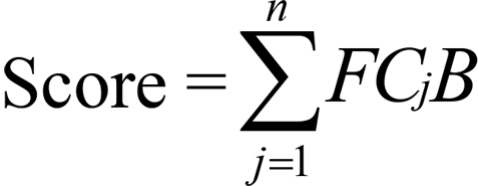


where *j* denotes particular feature (1 ≤ *j* ≤ *n*) among each epigenetic category (assuming *n* total features), *B* indicates whether the tested positive SNP was annotated (*B* = 1) or unannotated (*B* = 0) on feature *j*. Therefore, we can obtain 4 independent functional scores across 4 different epigenetic categories for each tested SNP. For each epigenetic category, we further scored for all negative SNPs to build null distribution, and derived the epigenetic functional support on each positive SNP if its functional score was higher than the top 5% ranked score value of all negative SNPs.

#### Allele-specific motif analysis.

We analyzed the allele-specific TF-binding motifs on all autoimmune positive SNPs using FIMO from MEME Suite toolkit (v4.11.0) (82) with default parameters, and TF motifs from 5 public motif databases, including JASPAR (2018 version) (83), HOCOMOCO (v11) (84), SwissRegulon (85), Transfac, and Jolma2013 (86). To identify potential functional motifs, we focused the motif search on TF genes expressed in at least 1 of the 20 blood immune cells from Roadmap (24) or DICE (40) (RPKM >1). The allele-specific binding motifs predicted by at least 2 different data sets were retained.

#### Molecular QTL association analysis.

We collected different molecular QTL data in multiple blood cell types from 8 different studies (28–31, 87–90) (Supplemental Table 3), including bQTL on 5 immune-relevant TFs (NF-κB, PU.1, Stat1, JunD, and Pou2f1) (28), hQTL (H3K4me1/H3K4me3/H3K27ac) (28, 29, 87, 88), dsQTL (30, 87), and caQTL (31, 89, 90). For all QTL data sets, the tested SNP and molecular peak (TFBSs or ChIP-Seq peaks) pairs could be divided into either local (SNP located within molecular peak) or distal ones (SNP located beyond molecular peak). We retained significant association results between autoimmune positive SNPs and local molecular peaks passed multiple testing corrections (FDR < 0.1).

#### Functional SNP prioritization.

Any autoimmune-positive SNP with both functionality support by at least 1 epigenetic category in the functional scoring and predicted allele-specific regulatory activity to be potential functional.

### Predicting target genes for prioritized functional SNPs

#### cis-QTL analysis.

We examined the cis-QTL association between prioritized noncoding SNPs and all nearby genes in 1 M region. We collected 12 cis-eQTL and 2 cis-pQTL data over 20 blood immune cell types from 13 different published studies (Supplemental Table 6). For pQTL data from the INTERVAL study (91), we extracted all cis-pQTL (1 M surrounding gene TSS) pairs and transformed the protein ID to gene symbol ID using the UniProt online tools. For any full QTL data set without multiple testing corrections, we adjusted the original *P* value using the FDR method. All significant QTL results with probe and/or gene level FDR <0.05 validated by at least 2 different data sets were retained.

#### 3D chromatin interaction analysis.

All SNP-gene pairs with cis-QTL associations were divided into either local (SNPs within target gene promoter, 1 kb surrounding TSS) or distal (SNPs beyond target gene promoter) genes. We collected chromatin interaction assay (5C, in situ Hi-C, capture Hi-C, HiChIP, and ChIA-PET) and predicted chromatin interaction data (IM-PET, PreSTIGE, PHM) on multiple blood immune cell types from 11 different studies (Supplemental Table 6). To validate the long-range regulation between distal SNP-gene pairs, the 3D chromatin interactions between prioritized SNP and gene transcript promoter region (GENCODE v19) were examined using bedtools v2.25.0 (81). The integration of cis-QTLs and 3D chromatin interactions may better identify causal regulatory effect at GWAS loci by diminishing the potential accidental overlapping with QTLs for GWAS SNPs. All distal SNP-gene pairs with chromatin interaction evidence from at least 2 different data sets were retained.

#### Colocalization analysis.

To validate the potential causal genetic regulatory effect for filtered target genes, we employed 2 complementary methods (32, 33) to assess whether the detected GWAS signal and cis-QTL association shared the same causal variant. For the available 16 GWAS summary and 7 full QTL data sets (Supplemental Tables 1 and 6), we used the Coloc (32) method using the coloc R package for colocalization analysis. The Coloc method (32) adopted a Bayesian statistical test using summary-level data to estimate 5 posterior probabilities: no association with either GWAS or QTL (PP0), association with GWAS while not with QTL (PP1), association with QTL while not with GWAS (PP2), association with GWAS and QTL while with 2 independent SNPs (PP3), association with both GWAS and QTL with one shared causal SNP (PP4). We defined the 100 kb region surrounding each GWAS index SNP (*P* < 5×10^–8^) and tested for colocalization with any overlapping QTL genes. For all curated GWAS and QTL data sets (including data sets with no full summary-level data available, Supplemental Tables 1 and 6), we used PICCOLO (33), another adapted Coloc method, for colocalization analysis. PICCOLO (33) estimates the colocalization of GWAS and QTL Probabilistic Identification of Causal SNPs (PICS) (17) credible set using reported lead SNPs and *P* value. PICS is a fine-mapping algorithm used to estimate the probability of each SNP being causal at a given locus (17). We performed PICCOLO analysis as described by Tachmazidou et al. (92). Briefly, we estimated the PICS credible set for each lead GWAS index SNP and each top QTL SNP using pics.download. Then, we performed colocalization analysis using pics.coloc.lite with a default parameter. For both Coloc and PICCOLO, any genes with both PP4 greater than 80% and significant QTL association with prioritized SNPs from at least 2 cis-QTL data sets were considered to support colocalization.

#### Local and distal target gene prediction.

We defined target gene, exclusively regulated by functional SNPs within its promoter region (1 kb surrounding TSS), as a local gene, and any other gene that could be regulated by distal functional SNPs as a distal gene. We predicted local or distal target genes on prioritized SNPs using different strategies. For local ones, any genes with both cis-QTLs association and colocalization evidence were prioritized to be potential target genes. For distal ones, any genes with cis-QTLs association, 3D chromatin interaction, and colocalization were considered to be potential target genes.

### Comparison with other functional scoring Methods

#### Curation of top-ranked SNPs.

We compared our SNP prioritization method with 5 other functional scoring methods, including 3DSNP (12), FIRE (10), GWAS4D (13), IW-Scoring (14), and RegulomeDB (11). Prioritized functional SNPs under different minimum epigenetic functionality evidence by our method (≥4, ≥3, ≥2, and ≥1, Supplemental Table 4) were extracted for functional comparison with equivalent or approximately equivalent top-ranked SNPs by other compared methods (see Supplemental Methods for a detailed description).

#### Functional enrichment comparison.

For collected functional SNPs set from each methods, we firstly compared their experimentally validated SNPs count in 3 cell types (blood mononuclear cells, K562 and HepG2) from 2 recent high-throughput screen reports (35, 36). We next compared their functionality enrichment on multiple regulatory data support using Fisher’s exact test, including (a) SNPs with predicted local or distal target genes (see gene prediction method in detail), (b) SNPs annotated with molecular QTL (bQTL, hQTL, dsQTL and caQTL) on multiple blood immune cell types (Supplemental Table 3), (c) reported causal SNPs associated with 16 autoimmune diseases prioritized by the PICS approach (17), and (d) SNPs annotated with eRNA from FANTOM5 (37).

### Exploring immunologically related functions for predicted target genes

#### Pathway analysis and functional genes curation.

We performed biological pathway enrichment analysis (including GO, KEGG, DO and Reactome pathway) for all predicted gene targets using clusterProfiler R package with default parameter (43), except that setting use_internal_data = TURE for KEGG enrichment analysis to enable online query from the latest KEGG data. Multiple testing was corrected by the FDR method with significance level at FDR < 0.05. To identify potential immunologically related genes, we manually curated immunologically related biological pathways from all annotated terms on predicted target genes. We also collected immunologically related genes from other public data sets, including the IMPC portal (http://www.mousephenotype.org/, release-9.2), the OMIM)database (https://www.omim.org/), and the DisGeNET database (http://www.disgenet.org/home/, v6.0, expert curated or text mining predicted genes) (41). All data sets were downloaded or queried online in May 2019.

#### Gene expression and tissue-specific expression analysis.

We collected gene expression data on 5 blood immune cell types (CD4 memory, CD4 naive, Mobilized CD34, Peripheral blood mononuclear, and GM12878) from Roadmap (24) and 15 primary immune cells types from the DICE project (http://dice-database.org/) (40). Gene expression was measured by RPKM (reads per kilobase per million mapped reads). In addition,we collected the gene lists with tissue-specific expression (as based on a specificity index threshold [pSI], pSI < 0.01) in 25 broad GTEx tissue types based on a previous report by Wells et al. (39).

#### SMR analysis.

We analyzed the causal relationship between predicted target genes and autoimmune disease risk using 16 GWAS summary and 7 QTL summary data (Supplemental Tables 1 and 6) by the SMR approach (42). We ran SMR (v0.712) with default parameters. LD correlations between SNPs were estimated from 6743 unrelated European samples from the Atherosclerosis Risk in Communities (ARIC) data (dbGap accession phs000280.v3.p1.c1) (93) with one of each pair of individuals with a SNP-derived relatedness estimate of > 0.025 suggested by GCTA (v1.91) (94) randomly removed. Gene-disease pairs that passed both the multi-SNP-based SMR test (FDR adjusted *P*_SMR_ < 0.05) and the heterogeneity test by HEIDI (*P*_HEIDI_ > 0.05) were considered to be potential causal.

### Regulatory TF analysis

We performed enrichment analysis for all allele-specific binding motif TFs on functional autoimmune SNPs by comparing annotated functional SNPs with all positive autoimmune SNPs using Fisher’s exact test. For each TF with significant higher enrichment on autoimmune SNPs (Bonferroni’s adjusted *P* < 0.05, FC > 1), we assigned the predicted regulatory targets of its binding SNPs as its direct regulatory target genes. The TF-gene regulatory network was visualized by Cytoscape V3.4 (http://www.cytoscape.org/). Master TFs on 26 blood cell types are collected from a previous report (56).

### Drug target and drug repurposing analysis

#### Curation of drug target genes.

Clinically approved or experimental drug target genes with known indications were obtained from 3 different databases, including the DrugBank database (https://www.drugbank.ca/, v5.1.2) (57), the Therapeutic Target Database (TTD, 2018 updated) (58), and Open Targets database (59). All 3 drug databases were queried in March 2019. For the TTD data set, we translated the UniProt protein ID into corresponding gene symbol ID using UniProt online tools. All drug indications were manually classified into autoimmune diseases, immunologically related diseases (allergies, infections, inflammations, rejection, immune system diseases, and hematologic malignancies), or other diseases.

#### Curation of druggable genes.

We collected potentially druggable genes from either the drug-gene interaction database (DGIdb) (www.dgidb.org, v3.0.2) (60), Pharos (https://pharos.nih.gov/idg/targets) (61), or a previous report by Finan et al. (62). We queried DGIdb and Pharos in March 2019. DGIdb organized druggable genome under 2 classes, including more than 35 validated or predicted drug-gene interaction types from 20 disparate sources, and 39 gene categories associated with druggability. Pharos classified all targets into 4 groups by characterizing the degree to which they are not studied (labeled Tdark) or studied (labeled Tbio, Tchem, or Tclin). The studied targets from Pharos were retained. Any gene targets with druggability evidence from at least 2 resources were prioritized as potentially druggable.

#### Predicting new potential drug target genes.

For all annotated drug target or druggable genes, we analyzed protein-protein interaction (PPI) between these genes and all other genes. PPI was queried online from the STRING database (https://string-db.org/) in June 2019, with only high-confident interacted pairs (interaction score > 0.9) retained. By leveraging both PPI and upstream autoimmune disease regulatory information, we can prioritize new potential drug target gene A or for a particular disease, drug target gene B, by filtering whether (a) A has strong PPI (interaction score > 0.9) with any drug target gene C, which had known indication on autoimmune disease B; (b) both A and C are regulated by upstream functional SNPs predisposing to autoimmune disease B; and (c) A is either a known drug target gene or predicted druggable gene. The predicted genes with known indication on other diseases might suggest new potential drug repurposing opportunities.

### Data availability

All analysis results are available at http://fngwas.online Analysis pipeline scripts are available at https://github.com/xjtugenetics/fnGWAS

### Statistics

The χ^2^ test was used to determine epigenetic features with significantly more enrichment in autoimmune positive SNPs compared with background SNPs with significance level at Bonferroni’s adjusted *P* < 0.05. Multiple testing on cis-QTL data sets was corrected using FDR method by R with significance level at FDR < 0.05. Colocalization analysis was conducted using Coloc (32) and PICCOLO (33) with posterior probability PP4 greater than 80% considered to support the colocalization between GWAS and cis-QTL association. Motif analysis was conducted using FIMO from MEME Suite toolkit (v4.11.0) (82) with default parameters (*P* < 0.0001). Multiple testing on molecular QTL data sets was corrected using FDR method by R with significance level at FDR < 0.1. Functional enrichment for immune cell–associated regulatory data (motif and molecular QTL) on prioritized functional SNPs, as well as all collected immune-relevant functional data sets (IMPC, OMIM, SMR, DisGeNET, TSEA, gene expression, and drug target) on predicted target genes was analyzed using Fisher’s exact test with significance level at *P* < 0.05. Gene pathway enrichment analysis was performed using clusterProfiler R package (43) with significance level at FDR < 0.05. To identify potential causal GWAS effector genes, we ran SMR (v0.712) (42) with default parameters, with genes passing both the multi-SNP–based SMR test (FDR < 0.05) and heterogeneity test (*P*_HEIDI_ > 0.05) considered potential causal. Functional enrichment for motif TFs was compared between prioritized functional SNPs with predicted target genes and all autoimmune SNPs using Fisher’s exact test with significance level at Bonferroni’s adjusted *P* < 0.05. Functional enrichment for druggable or known drug target genes or genes with PPI was compared between predicted target genes, and all genome genes using Fisher’s exact test with significance level at *P* < 0.05.

## Author contributions

TLY and YG designed the study. XFC conducted the data analysis. XFC and YG wrote the manuscript with input from HNT. MRG, FJ, and CCL designed the website. YYD, HW, XRZ, and LZ contributed to the manuscript preparation. SSD contributed to the development of scoring approach. All authors read and approved the final manuscript.

## Supplementary Material

Supplemental data

Supplemental Tables 1-17

## Figures and Tables

**Table 1:**
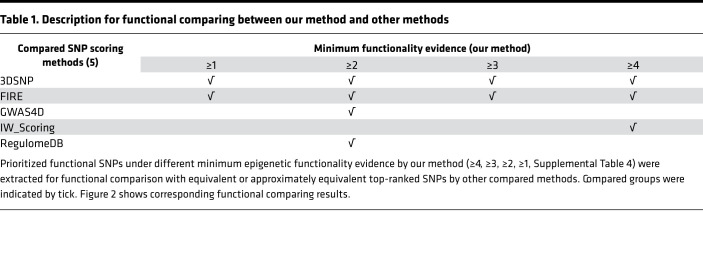
Description for functional comparing between our method and other methods

## Version 1 09/03/2020

Electronic publication
