# Supplementary material for: Multiomics dissection of molecular regulatory mechanisms underlying autoimmune-associated noncoding SNPs
Source: JCI Insight. 2020 Sep 3;5(17):e136477. doi: 10.1172/jci.insight.136477 (PMC7526455; doi:10.1172/jci.insight.136477)
Supplement: Supplemental data [file jciinsight-5-136477-s104.pdf]

## Supplemental Methods

### Comparison with other functional scoring methods

***Curation of top-ranked SNPs.*** We compared our SNP prioritization method with five other functional scoring methods, including 3DSNP (1), FIRE (2), GWAS4D (3), IW-Scoring (4) and RegulomeDB (5). The IW-Scoring (4) integrated eleven commonly used scoring methods to assign SNP a combined significance level (*P*-value) and outperformed any single method. We therefore did not compare our method with these eleven methods. Functional scores of all autoimmune positive SNPs from these methods were collected from online database in March 2019. We extracted prioritized autoimmune SNPs by our method under four different minimum functionality evidence (≥4, ≥3, ≥2, ≥1, n = 1,292 ~ 9,719, Table S4), and extracted equivalent or approximately equivalent top-ranked SNPs by other five methods for functional comparison. (1) Since both 3DSNP and FIRE adopted the quantitative scoring system, we selected those top scoring ranked SNPs equal to our prioritized SNPs under different minimum evidence (≥4, ≥3, ≥2, ≥1) for functional comparison, respectively. (2) The GWAS4D calculated combined regulatory probability (*P*-value) for examined variants by jointly considering cell type-specific regulatory potential and cell type-free composite score. We retained significant SNPs on GM12878 ($P < 0.01$, n = 4,838) for comparison with our prioritized SNPs under at least two functional evidence (≥1, n = 5,371), which had approximately equal SNP counts. (3) Similarly, we selected significant SNPs ($P < 0.05$, n = 341) by IW-scoring for functional comparison with our prioritized SNPs under at least four evidence (≥4, n = 1,292), which had the closest SNP counts. (4) The RegulomeDB adopted a category based scoring system (class from 1-7, with lower rank means higher functional support). We extracted SNPs ranked within class 1-3 (n = 5,156) for functional comparison with our prioritized SNPs under at least two functional evidence (≥2, n = 5,371), which had the closest SNP counts.

## Supplemental Figures

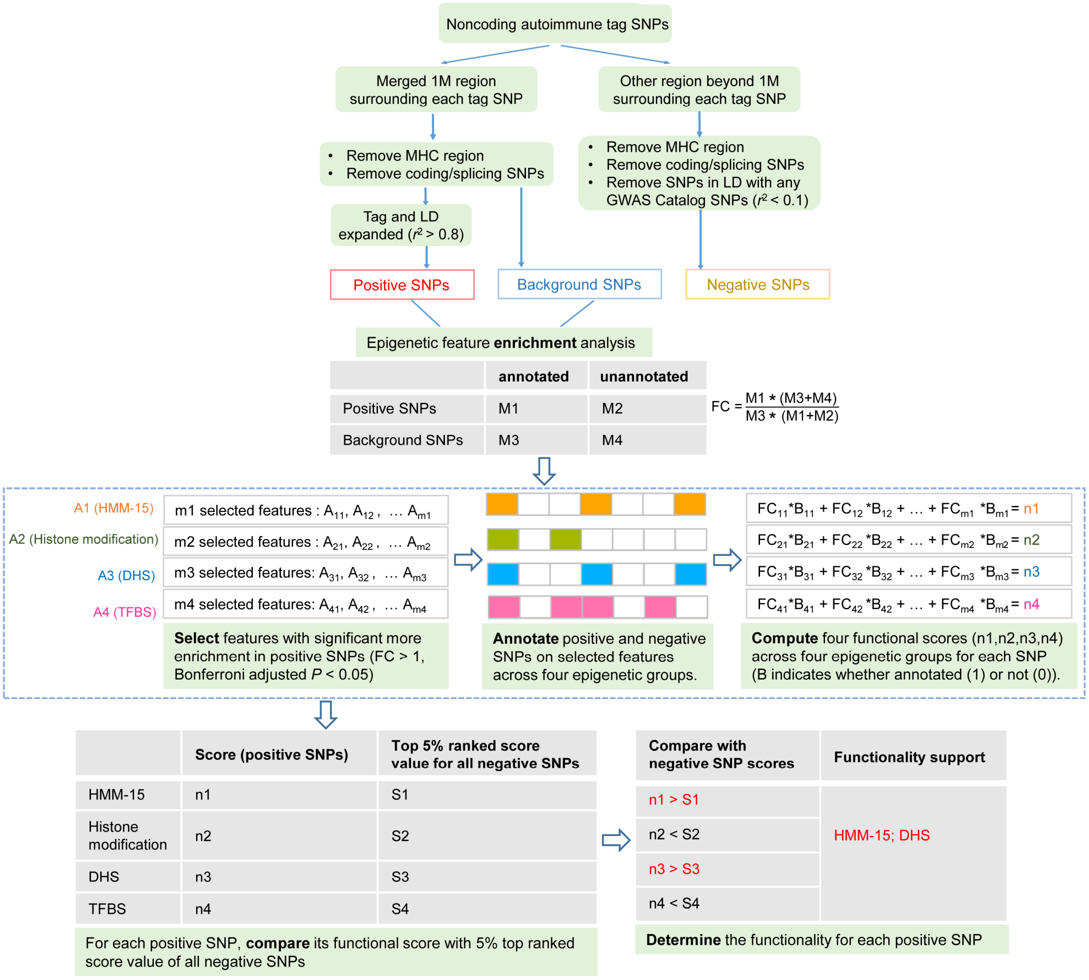


## Figure S1. Workflow of epigenetic functional scoring

The top panel shows definition for positive, background and negative autoimmune SNPs for the following epigenetic functional scoring. Any coding, spicing or major histocompatibility complex locus (MHC) region SNPs were removed. The middle panel shows the process for functional scoring. FC: fold enrichment. Epigenetic data in 47 blood immune cell types across four epigenetic categories (HMM-15, histone modification, DHS, TFBS) are used for enrichment analysis using Fisher's exact test (Table S2). M1-M4 denotes annotated or unannotated positive/background SNPs count on each epigenetic feature. A1-A4 denotes four epigenetic categories with m1-m4 significant enriched features for scoring. The bottom panel shows how to determine functionality support for each positive SNP. Each SNP had four scores (n1-n4) across four epigenetic groups, which were further compared with 5% top ranked score value of all negative SNPs (S1-S4) to determine its functionality support.

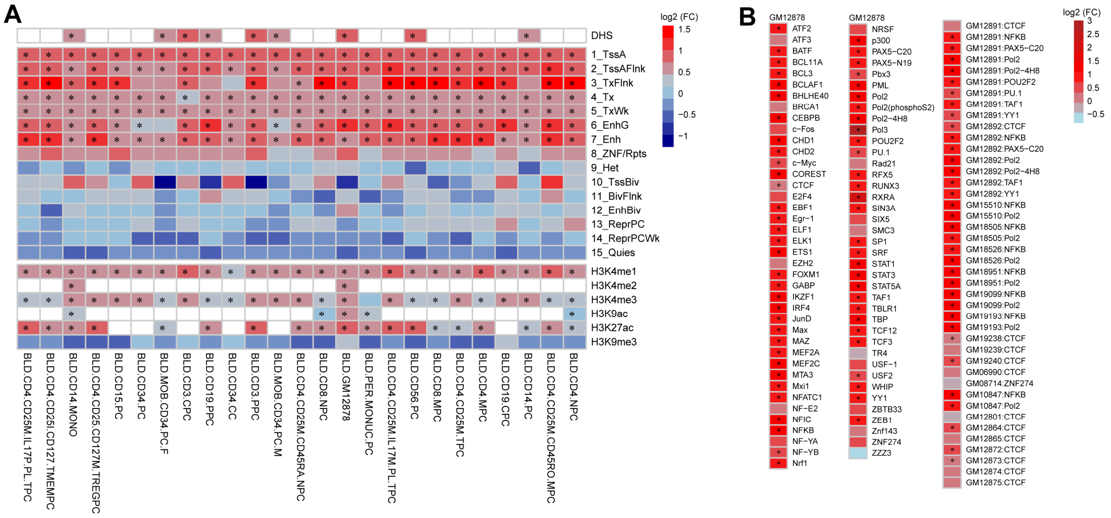


**Figure S2. Epigenetic enrichment analysis on autoimmune SNPs**

(A-B) Heatmap showing epigenetic feature enrichment analysis on 606 epigenetic data from 47 blood cell types across four epigenetic groups (Left: DHS, HMM-15, histone modification. Right: TFBS) between all autoimmune positive SNPs and background SNPs. FC: fold enrichment on each feature comparing autoimmune positive SNPs with background SNPs. Red color represents feature with higher enrichment in autoimmune positive SNPs (Log2FC > 0). All significant and active features (Bonferroni adjusted *P* < 0.05, FC > 1) were marked with asterisk and selected for next epigenetic functional scoring. Enrichment analysis was performed using Fisher's exact test. See detailed enrichment analysis results in Table S2.

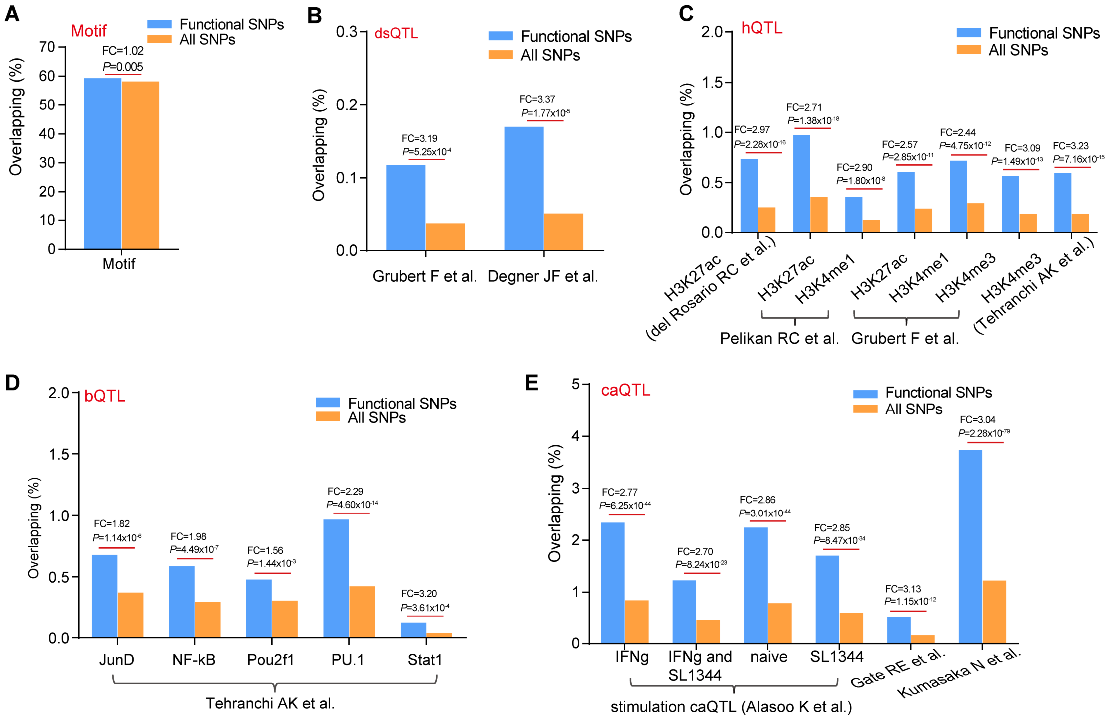


**Figure S3. Prioritized SNPs are significantly enriched in allele-specific motif and local and molecular QTLs**

(A-E) Functional enrichment for (A) allele-specific motif binding or (B-E) multiple intermediate molecular QTL data in multiple blood immune cell types (Table S3) on prioritized SNPs with epigenetic functionality support compared with all positive autoimmune SNPs. Multiple molecular QTL data are compared, including dsQTL (DNase-I hypersensitivity quantitative trait loci) (6,7), hQTL (histone modification quantitative trait loci) (6,8-10), bQTL (transcription factor binding quantitative trait loci) (10) and caQTL (chromatin accessibility quantitative trait loci) (11-13). Fisher's exact test was performed in A-E, with fold enrichment and *P*-value shown.

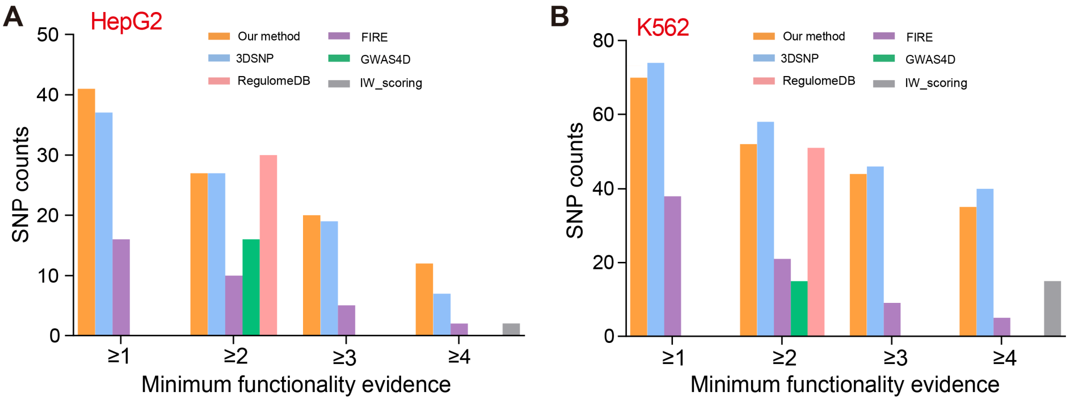


**Figure S4. Comparing integrative method with other methods using experimentally validated regulatory SNPs**

Comparison of experimentally validated functional SNPs between our integrative method and other five methods (1-5) from a high-throughput screen assay (14) in HepG2 cells (A) and K562 cells (B), respectively.

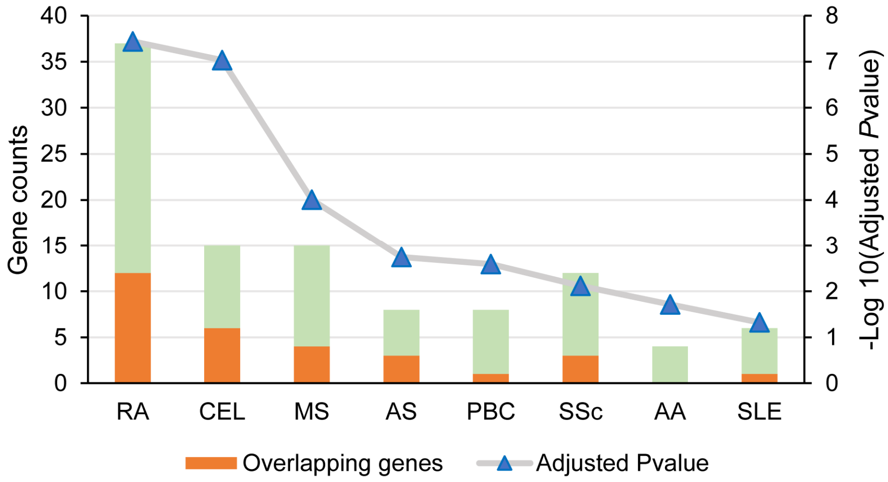


**Figure S5. Overlapping of autoimmune disease associated genes between significantly enriched disease gene pathways and 19 autoimmune diseases**

Among all significantly enriched autoimmune disease associated gene sets (FDR adjusted $P < 0.05$) from the Disease Ontology (DO) pathway enrichment analysis using clusterProfiler R package (15), 8 diseases overlapped with 19 autoimmune diseases analyzed by us. Both gene counts (histogram) from enriched pathway and significance level (line chart) was shown, with overlapping genes in each disease pathway regulated by functional SNPs associated with the same autoimmune disease marked by orange.

**Abbreviation:** RA: rheumatoid arthritis, CEL: celiac disease, MS: multiple sclerosis, AS: ankylosing spondylitis, PBC: primary biliary cirrhosis, SSc: systemic scleroderma, AA: alopecia areata, SLE: systemic lupus erythematosus.

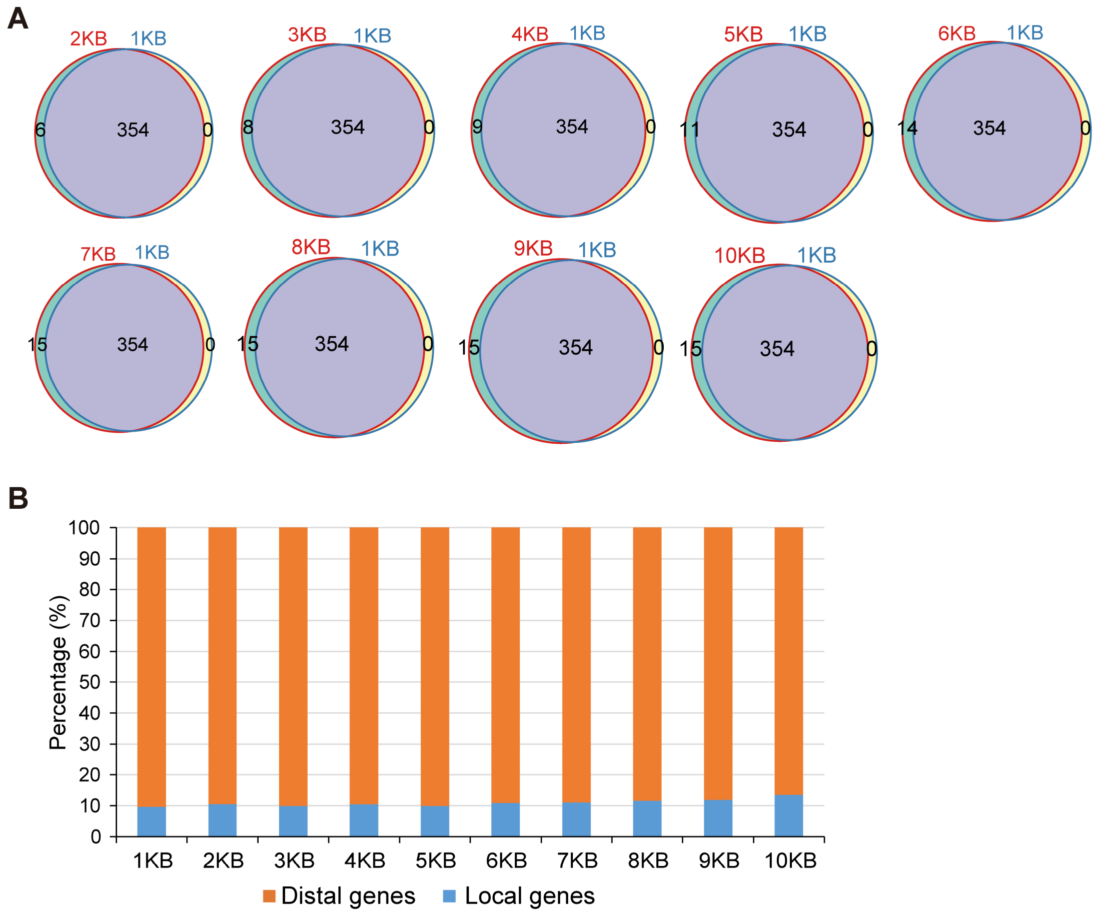


**Figure S6. Comparing target gene prediction results using different length of promoter definition**

(A) Venn diagrams showing overlapping of predicted target genes by defining promoter using different length of promoter definition (from 2-10KB surrounding transcription starting sites (TSS)) and more stringent definition (1KB surrounding TSS), which indicated negligible effect of length of promoter definition on target gene prediction results. (B) Comparing of percentage of predicted distal/local genes under different length of promoter definition (1-10KB surrounding TSS), which indicated negligible effect of length of promoter definition on dominant percentage of predicted distal genes.

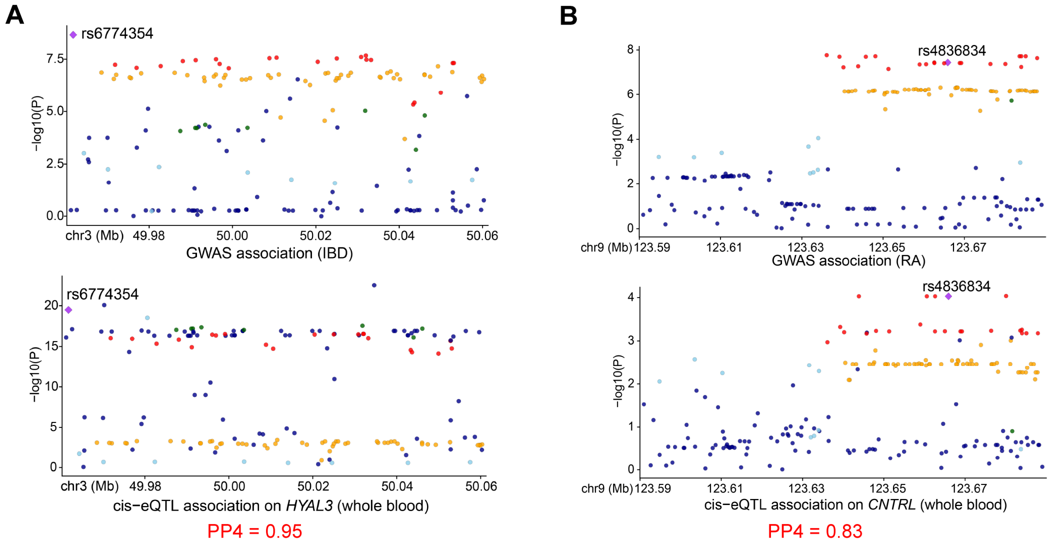


**Figure S7. Colocalization between GWAS association and cis-eQTL association on two example genes**

(A-B) Scatter plot showing comparison between GWAS association on selected autoimmune disease (upper) and cis-eQTL association on (A) *HYAL3* or (B) *CNTRL* in whole blood (below). Association signal within 100-KB surrounding the GWAS index ($P < 5\times10^{-8}$, Table S1) was shown. Co-localization analysis (16) was performed to validate the potential causal genetic regulatory effect on autoimmune disease for these two genes. The posterior probability PP4 (the detected GWAS signal and cis-QTL association shared the same causal variant) was shown below. See Table S8 for all colocalization results.

**Abbreviation:** RA: rheumatoid arthritis, IBD: inflammatory bowel disease.

**A**

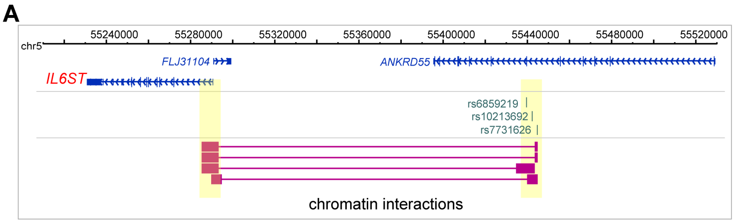


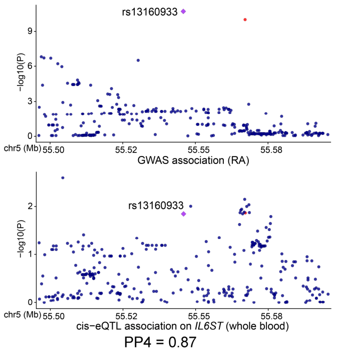


| Gene | Gene_expression | Causal genes (SMR) | DisGeNET | Pathway |
|---|---|---|---|---|
| *IL6ST* | expressed in 20 blood cells (RPKM >1) | √ | √ | 81 immunologically relevant pathways (eg, Positive regulation of immune system process, Regulation of cytokine production) |

**B**

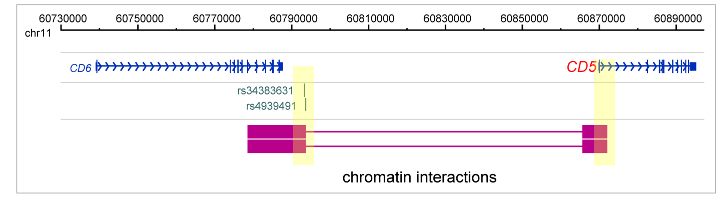


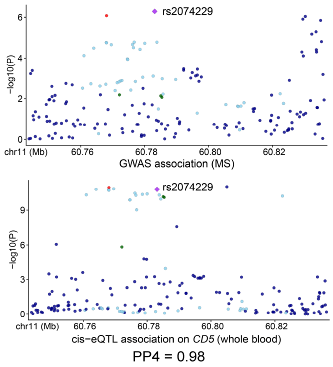


| Gene | Gene_expression | Cell specific expression | DisGeNET | IMPC | Pathway |
|---|---|---|---|---|---|
| *CD5* | expressed in 18 blood cells (RPKM >1) | pSI=0.0056 (blood) | √ | enlarged spleen; abnormal spleen morphology | 26 immunologically relevant pathways (eg, Immune system process, T cell aggregation) |

**Figure S8. Two example genes with known immunological roles exclusively regulated by distal functional SNPs**

Genomic annotation and chromatin interaction between distal functional SNPs and regulatory genes in (A-B) were visualized using WashU Epigenome Browser. Selected colocalization result on each gene are shown in right (see all cis-eQTL results in Table S5 and all colocalization results in Table S6). Summary of immunological roles on each gene are shown below (see detailed gene annotation results in Table S11). Both example genes in (A-B) had known immunological roles. Specifically, *IL6ST* encodes a receptor of IL-6 and its loss of mutation causes immunodeficiency and abnormal inflammatory responses (17). *CD5* is a well-known negative regulator of TCR and BCR signaling with critical roles in protecting against autoimmunity (18).

**Abbreviation:** RA: rheumatoid arthritis, MS: multiple sclerosis. IMPC: Gene KO in mouse displayed abnormal immune system phenotypes from the International Mouse Phenotyping Consortium (IMPC) portal (http://www.mousephenotype.org/) (19). expert curated or text mining predicted immune system diseases associated genes from the DisGeNET database (http://www.disgenet.org/home/) (20). SMR: causal effecter genes on autoimmune diseases identified by SMR (summary data–based Mendelian randomization) analysis (21).

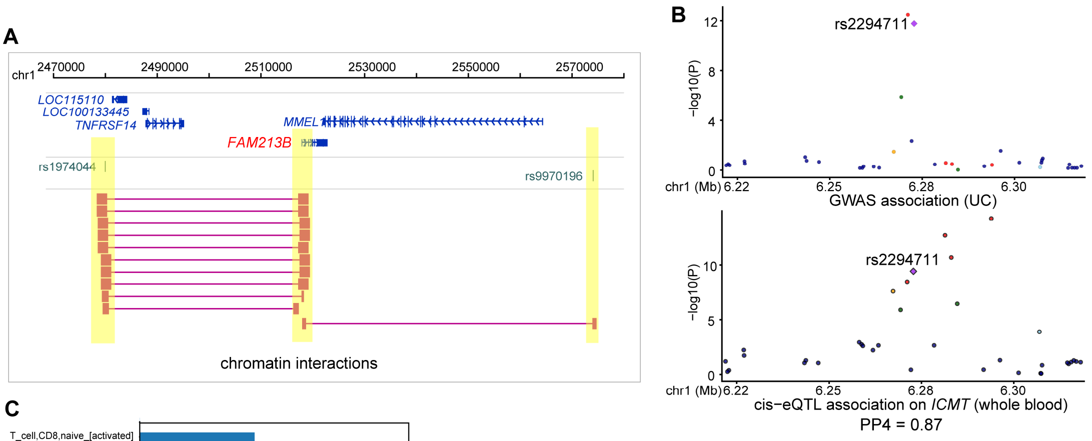


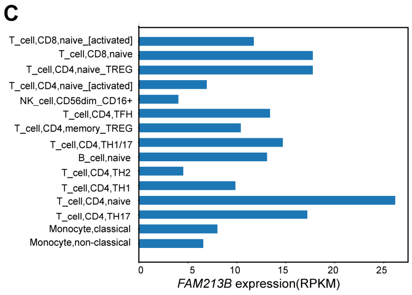


D

| Gene | Disease | QTL.dataset | topSNP | b_SMR | p_SMR | p_SMR_multi | p_HEIDI | FDR |
|---|---|---|---|---|---|---|---|---|
| FAM213B | IBD | eQTLGen_cis-eQTL | rs55658746 | 0.21 | 3.96E-06 | 5.68E-06 | 0.40 | 1.15E-03 |
| FAM213B | UC | eQTLGen_cis-eQTL | rs55658746 | 0.31 | 8.19E-08 | 1.69E-09 | 0.26 | 1.40E-06 |
| FAM213B | IBD | GTExV7_Whole_Blood | rs4648648 | 0.37 | 9.40E-05 | 6.99E-05 | 0.78 | 8.72E-03 |
| FAM213B | UC | GTExV7_Whole_Blood | rs4648648 | 0.53 | 2.80E-05 | 1.25E-05 | 0.61 | 2.18E-03 |
| FAM213B | PSC | GTExV7_Whole_Blood | rs4648648 | 0.58 | 3.09E-04 | 8.16E-05 | 0.32 | 9.90E-03 |

**Figure S9. Example gene (*FAM213B*) with unknown immunological roles exclusively regulated by distal functional SNPs**

(A) Genomic annotation and chromatin interactions between distal functional SNPs and *FAM213B* were visualized using WashU Epigenome Browser. (B) Selected colocalization result between *FAM213B* cis-eQTL association and GWAS association on ulcerative colitis (UC) are shown (see all cis-eQTL results in Table S5 and all colocalization results in Table S6). (C-D) Indicative immunological relevant function on *FAM213B* was shown, including (C) expression in multiple blood cells and (D) causal effect on several autoimmune diseases identified by SMR analysis (21).

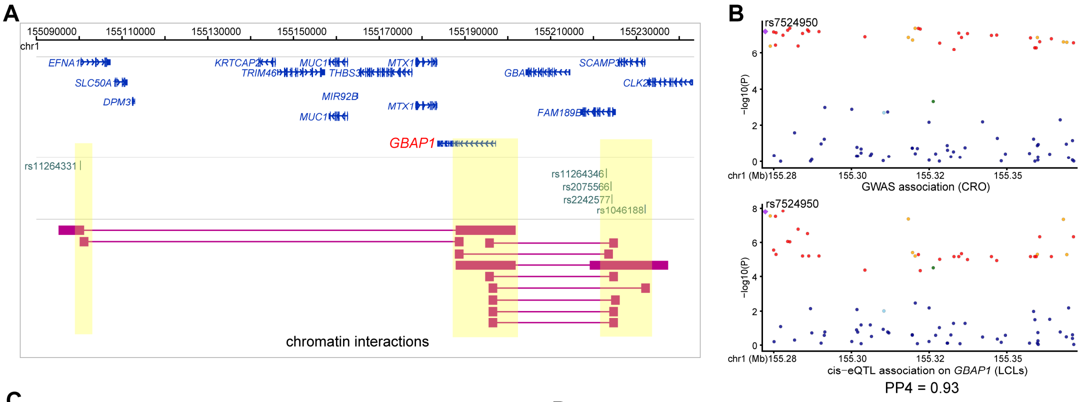


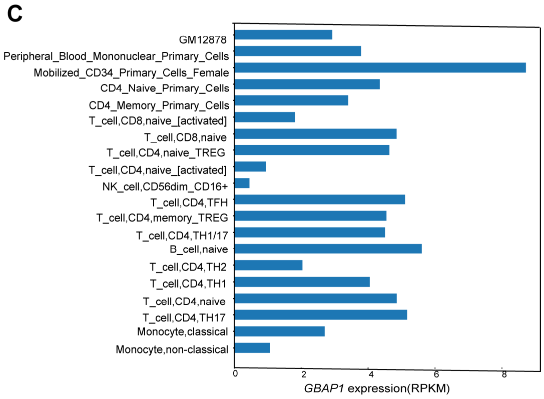


**D**

| Gene | Disease | QTL.dataset | topSNP | b_SMR | p_SMR | p_SMR_multi | p_HEIDI | FDR |
|---|---|---|---|---|---|---|---|---|
| GBAP1 | CRO | GTExV7_LCLs | rs2974929 | -0.13 | 2.16E-04 | 4.67E-05 | 0.29 | 6.31E-03 |
| GBAP1 | IBD | GTExV7_LCLs | rs2974929 | -0.10 | 4.92E-05 | 2.73E-05 | 0.75 | 4.09E-03 |
| GBAP1 | CRO | GTExV7_Whole_Blood | rs2075570 | -0.23 | 1.84E-04 | 3.59E-04 | 0.88 | 2.98E-02 |
| GBAP1 | IBD | GTExV7_Whole_Blood | rs2075570 | -0.19 | 3.10E-05 | 1.21E-04 | 0.74 | 1.33E-02 |

**Figure S10. Example gene (*GBAP1*) with unknown immunological roles exclusively regulated by distal functional SNPs**

(A) Genomic annotation and chromatin interaction between distal functional SNPs and *GBAP1* were visualized using WashU Epigenome Browser. (B) Selected colocalization result between *GBAP1* cis-eQTL association and GWAS association on Crohn's disease (CRO) are shown (see all cis-eQTL results in Table S5 and all colocalization results in Table S6). (C-D) Indicative immunological relevant function on *GBAP1* was shown, including (C) expression in multiple blood cells and (D) causal effect on several autoimmune diseases identified by SMR analysis (21).

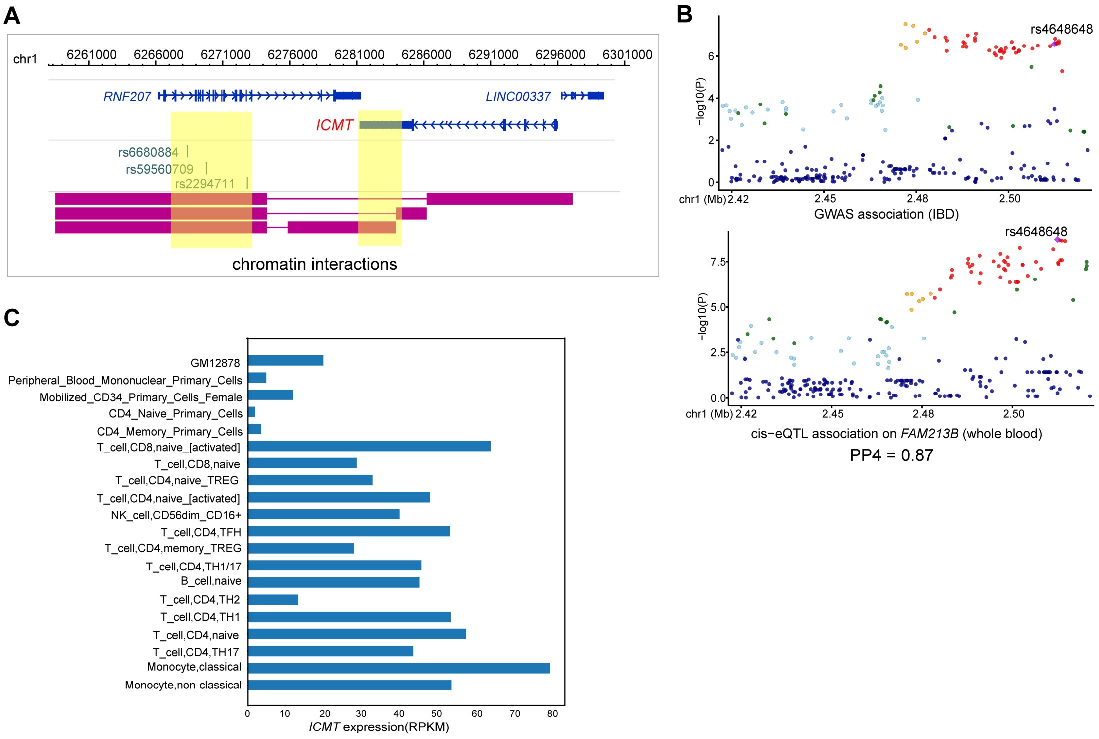


**Figure S11. Example gene (*ICMT*) with unknown immunological roles exclusively regulated by distal functional SNPs**

(A) Genomic annotation and chromatin interaction between distal functional SNPs and *ICMT* were visualized using WashU Epigenome Browser. (B) Selected colocalization result between *ICMT* cis-eQTL association and GWAS association on inflammatory bowel disease (IBD) are shown (see all cis-eQTL results in Table S5 and all colocalization results in Table S6). (C) Indicative immunological relevant function on *ICMT* was shown (expressed in multiple blood cells).

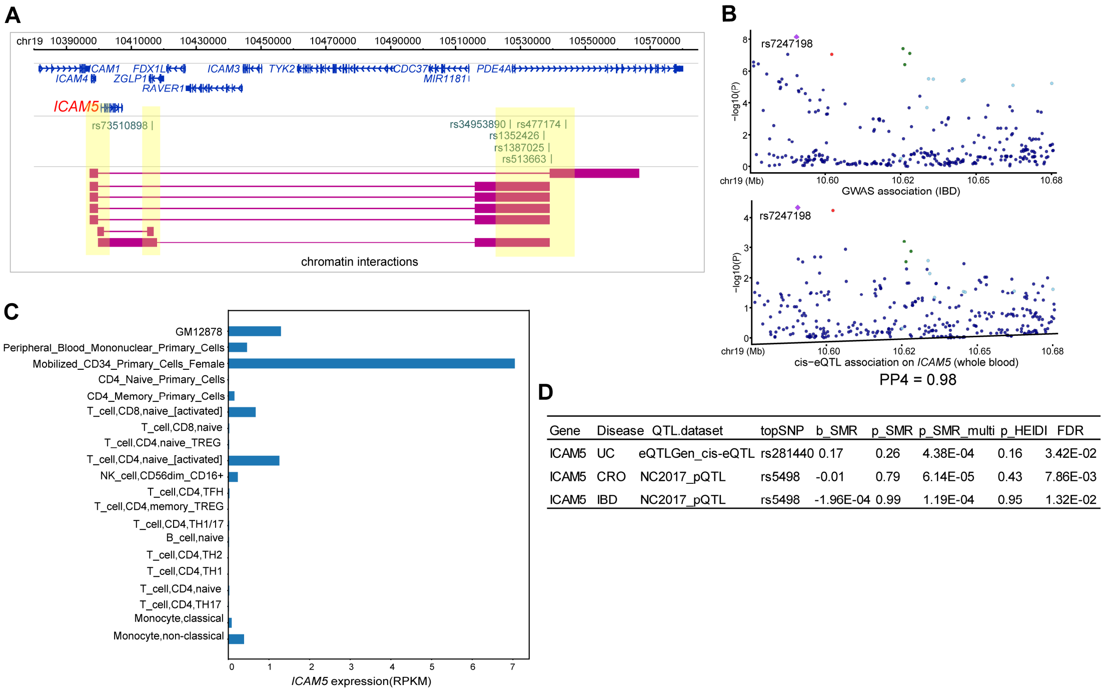


| Gene | Disease | QTL.dataset | topSNP | b_SMR | p_SMR | p_SMR_multi | p_HEIDI | FDR |
|---|---|---|---|---|---|---|---|---|
| ICAM5 | UC | eQTLGen_cis-eQTL | rs281440 | 0.17 | 0.26 | 4.38E-04 | 0.16 | 3.42E-02 |
| ICAM5 | CRO | NC2017_pQTL | rs5498 | -0.01 | 0.79 | 6.14E-05 | 0.43 | 7.86E-03 |
| ICAM5 | IBD | NC2017_pQTL | rs5498 | -1.96E-04 | 0.99 | 1.19E-04 | 0.95 | 1.32E-02 |

**Figure S12. Example gene (*ICAM5*) with unknown immunological roles exclusively regulated by distal functional SNPs**

(A) Genomic annotation and chromatin interaction between distal functional SNPs and *ICAM5* were visualized using WashU Epigenome Browser. (B) Selected colocalization result between *ICAM5* cis-eQTL association and GWAS association on inflammatory bowel disease (IBD) are shown (see all cis-eQTL results in Table S4 and all colocalization results in Table S6). (C) Indicative immunological relevant function on *ICAM5* was shown, including (C) expression in multiple blood cells and (D) causal effect on several autoimmune diseases identified by SMR analysis (21).

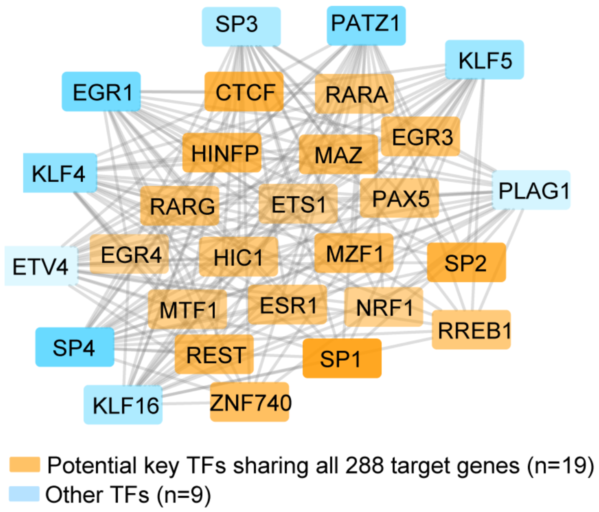


**Figure S13. Sharing of regulatory target genes between different significant TFs**

The orange rectangle represented 19 TFs sharing all target genes with another 9 TFs (blue), which might indicate their central regulatory roles. The transparency indicated counts of regulatory target genes on each TF.

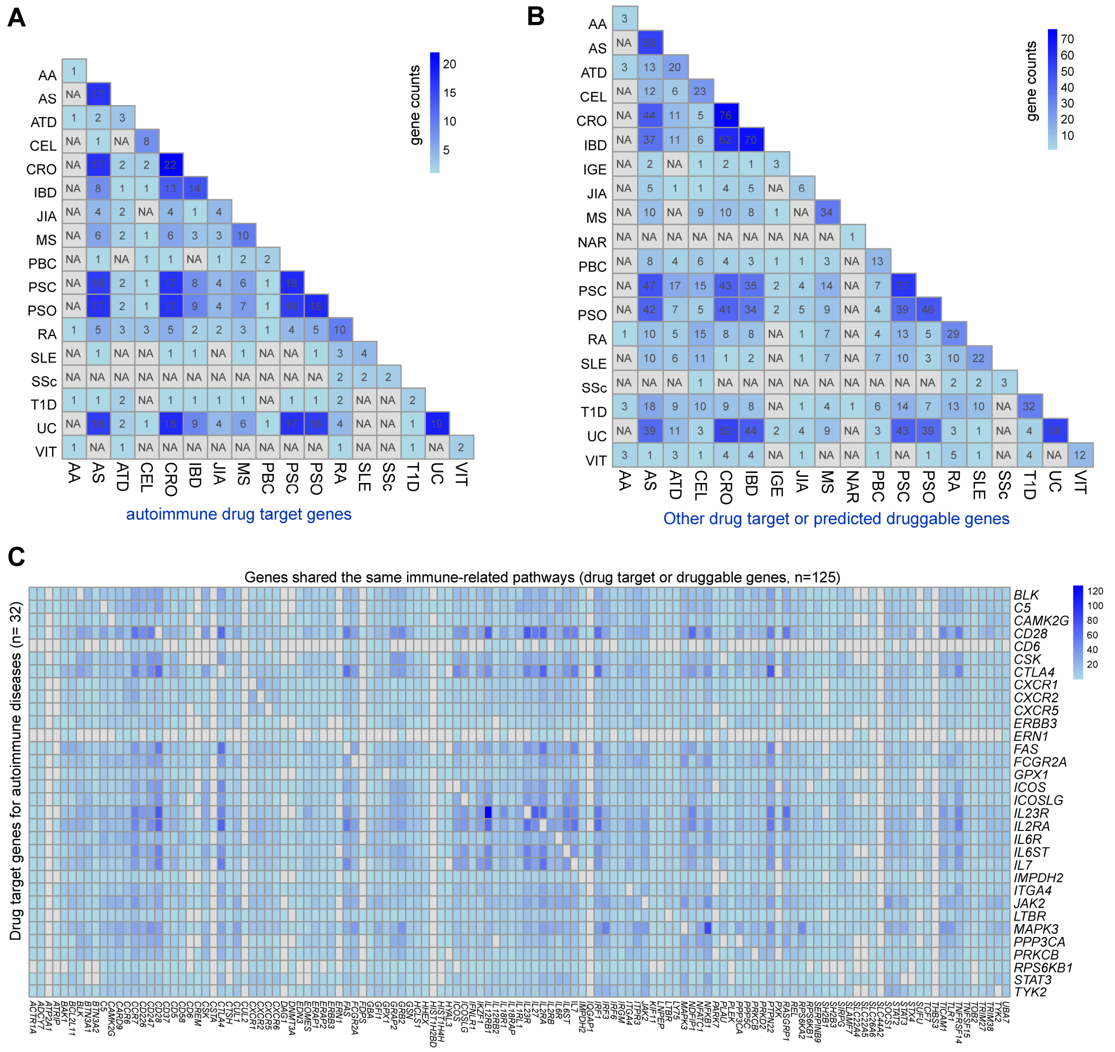


**Figure S14. Prevailing sharing of genetic disease-association and biological pathways on drug target genes**

(A-B) Count of (A) autoimmune drug target genes or (B) other drug target and predicted druggable genes associated with paired autoimmune diseases, with genes associated with individual disease shown in diagonal line. Disease association on gene targets are derived from their upstream functional SNPs (Table S5). (C) Counts of shared immunological related pathways between 41 known autoimmune-drug target genes (row) and all 198 drug target or druggable genes (column). Pathways were manually curated from all annotated biological terms (GO, KEGG, DO, Reactome) on predicted target genes (Table S10 and S11).

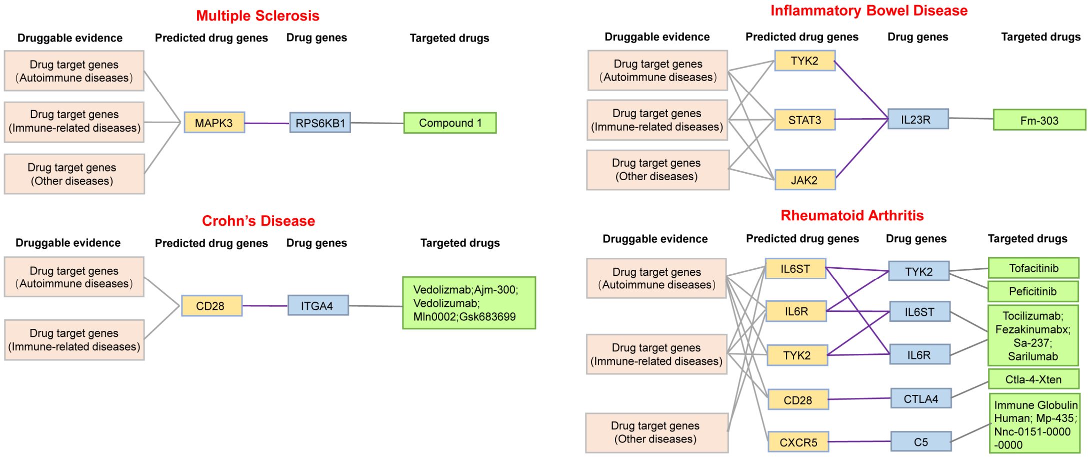


**Figure S15. Predicted new potential drug targets for four autoimmune diseases**

The yellow rectangle shows predicted new drug genes for four autoimmune diseases, which had strong PPI with known drug target genes (blue). All predicted drug genes had known indications on other autoimmune diseases or non-autoimmune diseases.

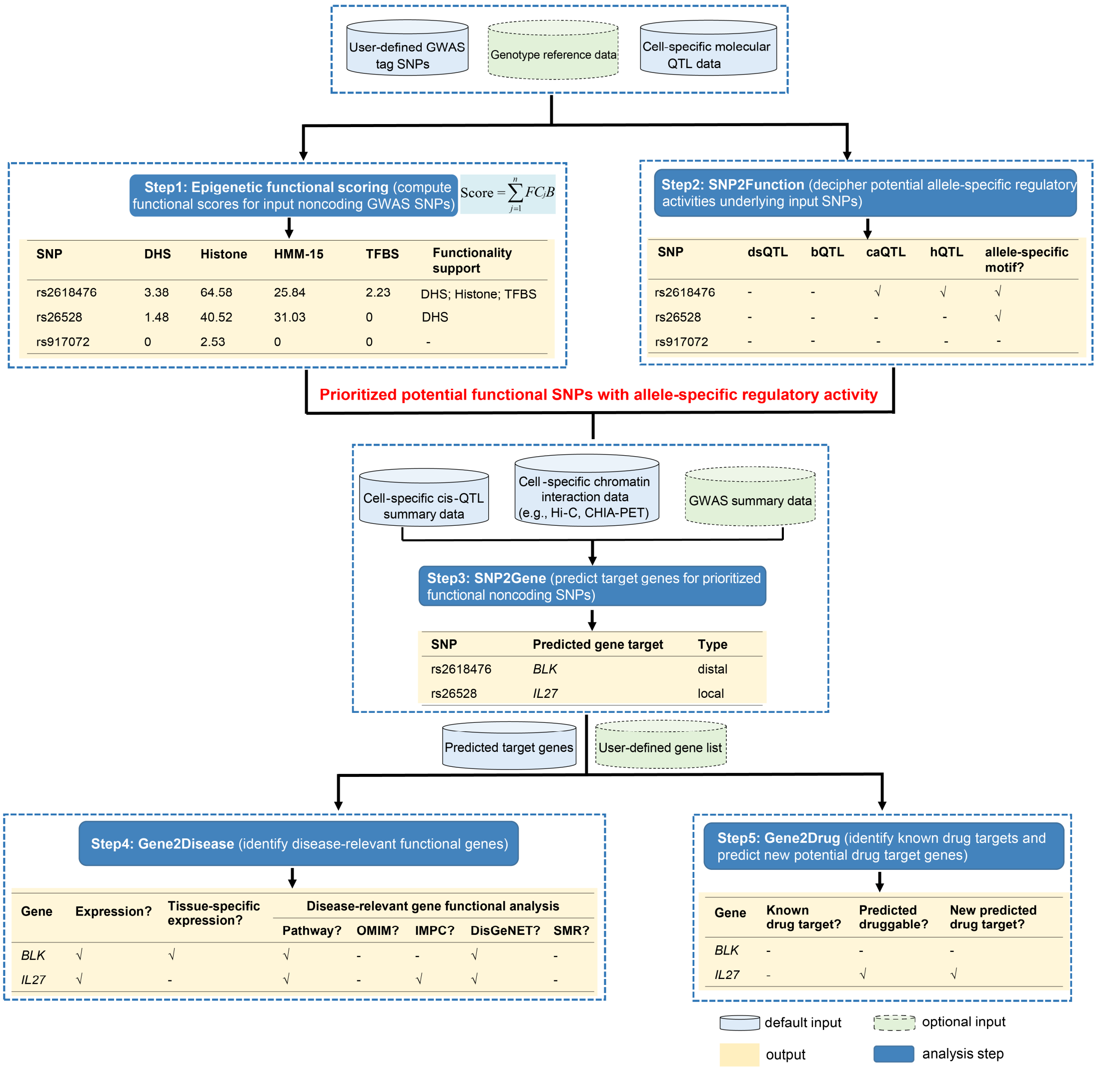


## Figure S16. Flowchart of fnGWAS pipeline

The blue rectangle summarized five main analysis steps of fnGWAS (dissecting the functionality of noncoding GWAS SNPs) pipeline, with aim for each step shown (Step 1-5). For each analysis step, the input data (represented by cylinder) and simplified example summarized output result (represented by yellow table) are shown, respectively. By default, fnGWAS begins with functional SNP prioritization by combing epigenetic functional scoring pipeline (Step1) and allele-specific analysis (Step2) using all susceptible SNPs associated with any interested diseases/traits as input, which outputs functional scores and functionality support for all positive SNPs (see detailed workflow for step 1 in Figure S1). Target gene prediction were then employed for all positive SNPs with functionality support (Step 3). Downstream functional analysis were then performed on predicted target genes (Step 4-5). Alternatively, each step of fnGWAS can be run independently, which support any user-defined input data. The fnGWAS have provided built-in 1000 genome v3 genotype data in European samples (22) for functional analysis. However, genotype data from population of any other ancestry (eg, European, African or Asian) was also applicable if user provided them. The whole pipeline including input annotation data are free available at https://github.com/xjtugenetics/fnGWAS or http://fngwas.online/download.php.

## Supplementary References


1. Lu Y, Quan C, Chen H, Bo X, Zhang C. 3DSNP: a database for linking human noncoding SNPs to their three-dimensional interacting genes. *Nucleic Acids Res.* 2017;45(D1):D643-D49.
2. Ioannidis NM, et al. FIRE: functional inference of genetic variants that regulate gene expression. *Bioinformatics.* 2017;33(24):3895-901.
3. Huang D, et al. GWAS4D: multidimensional analysis of context-specific regulatory variant for human complex diseases and traits. *Nucleic Acids Res.* 2018;46(W1):W114-W20.
4. Wang J, Dayem Ullah AZ, Chelala C. IW-Scoring: an Integrative Weighted Scoring framework for annotating and prioritizing genetic variations in the noncoding genome. *Nucleic Acids Res.* 2018;46(8):e47.
5. Boyle AP, et al. Annotation of functional variation in personal genomes using RegulomeDB. *Genome Res.* 2012;22(9):1790-7.
6. Grubert F, et al. Genetic Control of Chromatin States in Humans Involves Local and Distal Chromosomal Interactions. *Cell.* 2015;162(5):1051-65.
7. Degner JF, et al. DNase I sensitivity QTLs are a major determinant of human expression variation. *Nature.* 2012;482(7385):390-94.
8. del Rosario RC, et al. Sensitive detection of chromatin-altering polymorphisms reveals autoimmune disease mechanisms. *Nat Methods.* 2015;12(5):458-64.
9. Pelikan RC, et al. Enhancer histone-QTLs are enriched on autoimmune risk haplotypes and influence gene expression within chromatin networks. *Nat Commun.* 2018;9(1):2905.
10. Tehranchi AK, et al. Pooled ChIP-Seq Links Variation in Transcription Factor Binding to Complex Disease Risk. *Cell.* 2016;165(3):730-41.
11. Alasoo K, et al. Shared genetic effects on chromatin and gene expression indicate a role for enhancer priming in immune response. *Nat Genet.* 2018;50(3):424-31.
12. Gate RE, et al. Genetic determinants of co-accessible chromatin regions in activated T cells across humans. *Nat Genet.* 2018;50(8):1140-50.
13. Kumasaka N, Knights AJ, Gaffney DJ. High-resolution genetic mapping of putative causal interactions between regions of open chromatin. *Nat Genet.* 2019;51(1):128-37.
14. van Arensbergen J, et al. High-throughput identification of human SNPs affecting regulatory element activity. *Nat Genet.* 2019;51(7):1160-69.
15. Yu G, Wang LG, Han Y, He QY. clusterProfiler: an R package for comparing biological themes among gene clusters. *Omics.* 2012;16(5):284-7.
16. Giambartolomei C, et al. Bayesian test for colocalisation between pairs of genetic association studies using summary statistics. *PLoS Genet.* 2014;10(5):e1004383.
17. Spencer S, et al. Loss of the interleukin-6 receptor causes immunodeficiency, atopy, and abnormal inflammatory responses. *The Journal of experimental medicine.* 2019;216(9):1986-98.
18. Dalloul A. CD5: a safeguard against autoimmunity and a shield for cancer cells. *Autoimmun Rev.* 2009;8(4):349-53.
19. Rozman J, et al. Identification of genetic elements in metabolism by high-throughput mouse phenotyping. *Nat Commun.* 2018;9(1):288.
20. Piñero J, et al. DisGeNET: a comprehensive platform integrating information on human disease-associated genes and variants. *Nucleic Acids Research.* 2017;45(D1):D833-D39.
21. Zhu Z, et al. Integration of summary data from GWAS and eQTL studies predicts complex trait gene

targets. *Nat Genet.* 2016;48(5):481-87.
22. Sudmant PH, et al. An integrated map of structural variation in 2,504 human genomes. *Nature.* 2015;526(7571):75-81.
